# Assessing the hospital volume-outcome relationship in surgery: a scoping review

**DOI:** 10.1186/s12874-021-01396-6

**Published:** 2021-10-09

**Authors:** Mathieu Levaillant, Romaric Marcilly, Lucie Levaillant, Philippe Michel, Jean-François Hamel-Broza, Benoît Vallet, Antoine Lamer

**Affiliations:** 1 Univ. Lille, CHU Lille, ULR 2694 - METRICS: Évaluation des technologies de santé et des pratiques médicales, F-59000 Lille, France; 2 Methodologic and Biostatistics Department, CHU Angers, University Angers, 4 rue Larrey, F-49000 Angers, cedex 9 France; 3 Inserm, CIC-IT 1403, F-59000 Lille, France; 4 Department of Paediatric Endocrinology and Diabetology, Angers University Hospital, Angers, France; 5 Hospices Civils de Lyon ; Université Claude Bernard Lyon 1, Research on Healthcare Performance (RESHAPE), INSERM U1290, Lyon, France

**Keywords:** Volume-outcome relationship, Hospital, Quality indicator, Surgery

## Abstract

**Introduction:**

Many recent studies have investigated the hospital volume-outcome relationship in surgery. In some cases, the results have prompted the centralization of surgical activity. However, the methodologies and interpretations differ markedly from one study to another. The objective of the present scoping review was to describe the various features used to assess the volume-outcome relationship: the analyzed datasets, study population, outcome, covariates, confounders, volume modalities, and statistical methods.

**Methods and analysis:**

The review was conducted according to a study protocol published in *BMJ Open* in 2020. Two authors (both of whom had helped to design the study protocol) screened publications independently according to the title, the abstract and then the full text. To ensure exhaustivity, all the papers included by each reviewer went through to the next step.

**Interpretation:**

The 403 included studies covered 90 types of surgery, 61 types of outcome, and 72 covariates or potential confounders. 191 (47.5%) studies focussed on oncological surgery and 37.8% focussed visceral or digestive tract surgery. Overall, 86.6% of the studies found a statistically significant volume-outcome relationship, although the findings differed from one type of surgery to another. Furthermore, the types of outcome and the covariates were highly diverse. The majority of studies were performed in Western countries, and oncological and visceral surgical procedures were over-represented; this might limit the generalizability and comparability of the studies’ results.

**Supplementary Information:**

The online version contains supplementary material available at 10.1186/s12874-021-01396-6.

## Introduction

The hospital volume-outcome relationship in surgery has been extensively studied over the last decade. A significant relationship has been evidenced for various surgical procedures [1–4]; in all cases, a higher operating volume was associated with better patient outcomes. Given the consistency of this relationship from one setting to another, some researchers have recommended the creation of minimum volume thresholds in order to limit the number of centres with low levels of activity [5–7]. This recommendation is also in line with the guidelines issued by the Expert Panel on Weight Loss Surgery [8]. These research findings prompted the French health authorities to consider the establishment of thresholds for oncological surgery in 2007 [9].

Even though the volume-outcome relationship appears to be relevant for a variety of surgical procedures and has prompted greater centralization [10], Morch et al.’s recent systematic review highlighted marked methodological differences between the studies in this field and suggested that further research should focus on the features used to assess the volume-outcome relationship [4]. These methodological disparities have been confirmed in a few publications; the significance of the volume-outcome relationship may depend on the way the outcome was explored, the covariates included in the model, the qualitative or quantitative categorization of the volume, and/or the type of statistical test applied [11, 12]. Hence, a study’s methodology can have a direct impact on its conclusion [13, 14].

Most studies of the volume-outcome relationship have assessed mortality as the primary indicator. Although this is commonly assumed to be an essential outcome, mortality alone might not be sufficient for setting thresholds on surgical activity or for closing down low-volume centres - decisions that can have dramatic impacts on inequalities in health status and access to care [15]. In contrast, the potential lack of a significant relationship with volume does not mean that mortality is not of relevance for policy makers; it is acknowledged that this variable is positively associated with the length of hospital stay [16], recovery time [17], cost of the stay [18], related morbidity [19, 20] and (for cancer surgery) disease-free survival [21, 22]. Lastly, the identification of a positive volume-outcome relationship may not be enough to set thresholds. This doubt limits the reliability of this information as a basis for decision-making and the potential modification of organizational structures.

The above observations prompted us to consider that the volume-outcome relationship should be investigated more broadly. The objective of the present scoping review was to describe features that can be used to assess the volume-outcome relationship: the type of data analyzed, the study population, the study outcomes, the covariates and confounders considered, the hospital volume, and the interpretation of the results. Hence, this review of the volume-outcome relationship is intended to help researchers to choose outcomes and covariates of interest or even to identify new variables for investigation. Ultimately, this overview might help policy makers to understand the abundancy of the scientific literature and the breadth of this issue [23, 24].

## Method

The present review’s methodology (including the search and selection strategies and the analysis steps) is described elsewhere [25]. The review was conducted in six stages, as proposed by Arksey and O’Malley [26] and as subsequently modified by Levac et al. [27]. The present report complied with the Preferred Reporting Items for Systematic Reviews and Meta-Analyses (extension for scoping reviews) [28]. The main research question was as follows: how is the hospital volume-outcome relationship assessed in the field of surgery?

Suitable publications were identified according to the methodology developed by the Joanna Briggs Institute [29] (Table 1).

**Table 1:** Inclusion and exclusion criteria for studies of the volume-outcome relationship in surgery

Population	Concept	Context	Types of sources
Inclusion criterion: studies of the hospital volume-outcome relationship in surgery, the methodology of which was described in sufficient detail for the extraction of all requisite data. Exclusion criterion: studies of surgeon-specific volume-outcome relationships.	Inclusion criterion: methods used to assess the hospital volume-outcome relationship in surgery. Exclusion criterion: studies in which (i) the hospital volume was a covariate in a model and (ii) the impact of volume per se was not apparent.	Inclusion criteria: all types of surgery, all types of patient-related outcome (length of stay, mortality, morbidity, cost, etc.), all countries. Exclusion criteria: none.	Inclusion criterion: primary quantitative research publications written in English and published after 2009. Exclusion criterion: studies with other designs.

The PUBMED and Scopus databases were searched with the query shown in Table 2.

**Table 2:** Keywords and query used to search PUBMED and SCOPUS

Database	Keywords and query
PUBMED	Keywords: Volume, outcome, hospital, surgery, surgical, mortality, morbidity, cost Query: (“Volume-outcome” OR “Volume-mortality” OR (“hospital volume” AND (“outcome” OR “mortality” OR “morbidity” OR “cost”))) AND (“surgery” OR “surgical”) AND “hospital” NOT “surgeon”[TITLE]
Scopus	Keywords: Volume, outcome, hospital, surgery, surgical, mortality, morbidity, cost Query: TITLE-ABS-KEY ((“Volume-outcome” OR “Volume-mortality” OR (“hospital volume” AND (“outcome” OR “mortality” OR “morbidity” OR “cost”))) AND “surgery” AND “hospital”) AND NOT TITLE(“surgeon”) AND (LIMIT-TO (PUBSTAGE, “final”)) AND (LIMIT-TO(DOCTYPE, “ar”)) AND (LIMIT-TO (LANGUAGE, “English”))

The publications were screened, selected and reviewed independently by two authors: a resident in public health (ML) and a medical informatics specialist (AL), both of whom had helped to draft the study protocol.

The literature was screened first by title and then by abstract, according to the inclusion and exclusion criteria (Table 3). Publications were included if they met all the inclusion criteria and none of the exclusion criteria. In each stage of the review, this method was tested on 10 publications. The two reviewers then met and checked that they agreed on the inclusion and exclusion decisions. All publications selected by either of the reviewers went through to the next step. The two reviewers’ selections were not compared at the end of the title or abstract screening steps.

**Table 3:** Inclusion and exclusion checklist

Criteria	Review Result
Inclusion criteria:	
Assessment of the hospital volume-outcome relationship in surgery	❒ Yes ❒ No
A precise description of the methodology (how the outcome was assessed, how the hospital volume was analyzed, and how the statistical analysis was performed).	❒ Yes ❒ No
Exclusion criteria:	
Surgeon-specific volume-outcome relationship only	❒ Yes ❒ No
Hospital volume used only as a covariate	❒ Yes ❒ No
Publication as systematic review, qualitative study, editorial, letter to the editor, comment, narrative report, or any format other than primary quantitative research	❒ Yes ❒ No

**Fig. 1:**
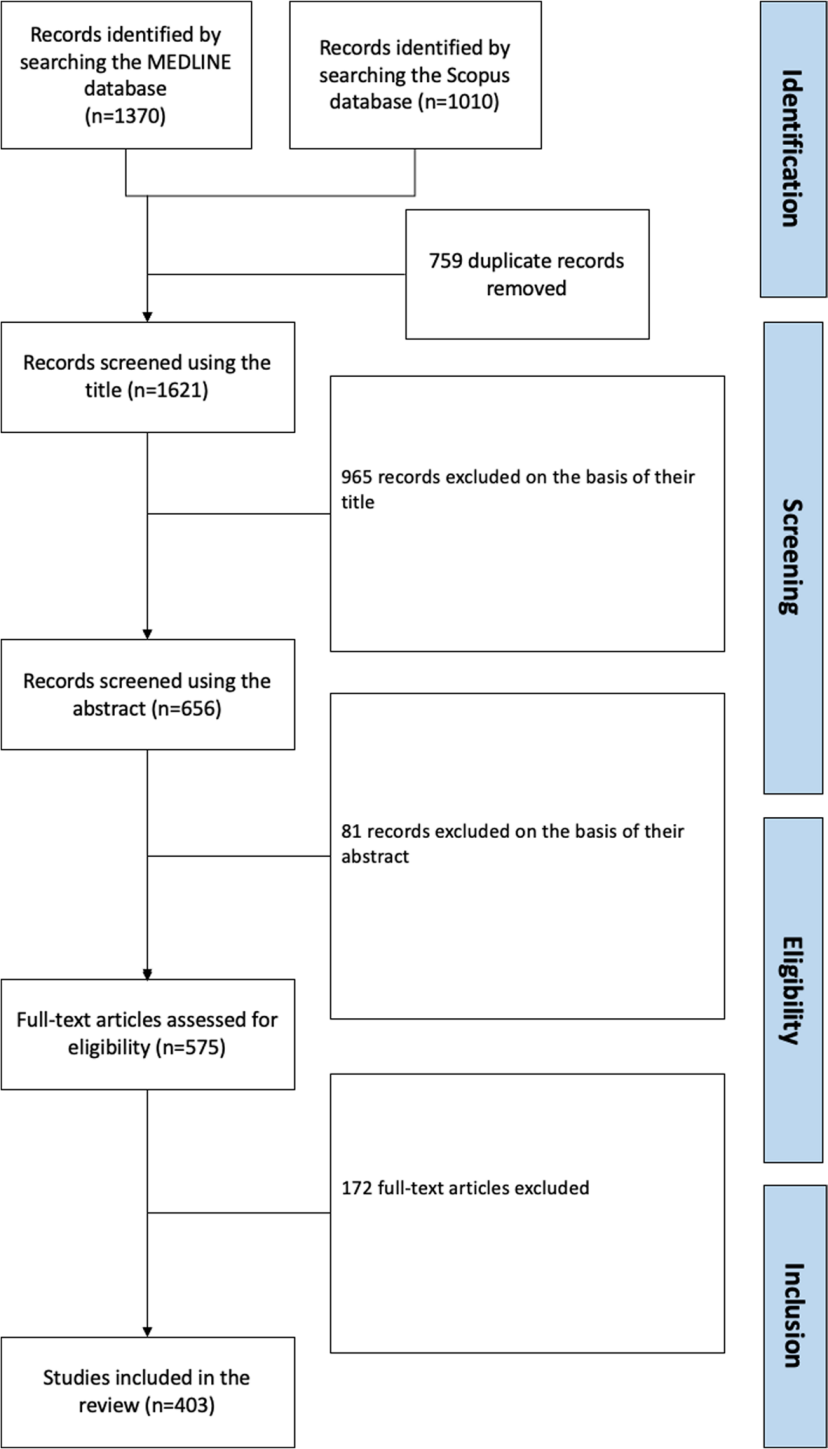
Flow chart

Lastly, the full text of selected publications were assessed for inclusion (Fig. 1). In the event of disagreement between the two reviewers, the final decision on inclusion was referred to a third reviewer (LL, who was also helped to design the study).

The reference lists of all selected publications were screened for additional studies meeting our inclusion and exclusion criteria.

The study data were extracted independently by the two reviewers, using a specific form (Supplementary Table 1). In the event of disagreement, the decision was referred to the third reviewer. After the data extraction form had been tested on the first 10 studies by both reviewers, it was validated as described in the study protocol [25]. No difficulties were encountered by either of the reviewers.

For each study, the following key data were extracted: first author, year of publication, country, study design, study objectives, the type of surgery, the database used to include patients, the inclusion and exclusion criteria, outcomes, confounders, statistical analyses, qualification of the volume variable, and conclusions. Using an inductive approach, the reviewers sorted the extracted data into the meta-categories listed in Table 4.

**Table 4:** The information extracted using the specific form, and references for each criterion

Type of data analyzed	Inclusion and exclusion criteria	Outcome	Confounders	Help in the results interpretation	Qualification of the volume variable	Conclusion of the study
The databases used were sorted into six categories, as recommended by Levac et al. [26]: electronic health records, administrative data, claims data, patient or disease registers, health surveys, or clinical trials data	The use (or not) of International Classification of Diseases (ICD) [30] to classify the diseases studied was recorded.	Outcomes were extracted and classified according to the categories typically observed in the literature: mortality, cost, length of stay, readmission, and others [4, 29–31]. The methods used to explore outcomes were detailed and then sorted using an inductive approach.	The scales or scores used to stratify analyses for the initial severity of the patient’s condition were classified as a known score (e.g. the Charlson Comorbidity Index (CCI), as adapted by Deyo [32, 33], or the Elixhauser score [34]) or another scale/score. All the covariates used to adjust the statistical models were listed and then sorted using an inductive approach.	The types of graphic representation of the results were classified as graphs, tables, or both. Graphs were subdivided into scatter plots, line graphs, bar graphs, histograms, pie charts, box plots or other graphs, as described by Slutsky et al. [28]	The variable used to assess hospital volume was classified as continuous, categorical, or both. If volume was considered as a categorical variable, we extracted more detailed information on the categories: quantiles, statistically defined cut-off points, arbitrary thresholds or those based on the literature, and other types.	Lastly, each study conclusion was noted and classified as having found (or not) a statistically significant positive (or negative) relationship between volume and outcome.

## Results

### Description of the publications included

We identified a total of 1010 publications in the Scopus database, and 1370 in the PUBMED database. After the removal of duplicates, 1621 publications remained. Next, 965 publications were excluded on the basis of the title, 81 were excluded on the basis of the abstract, and 172 were excluded on the basis of the full text (Fig. 1). No additional publications were included after screening the reference lists of those found in Scopus or PUBMED.

**Fig. 2:**
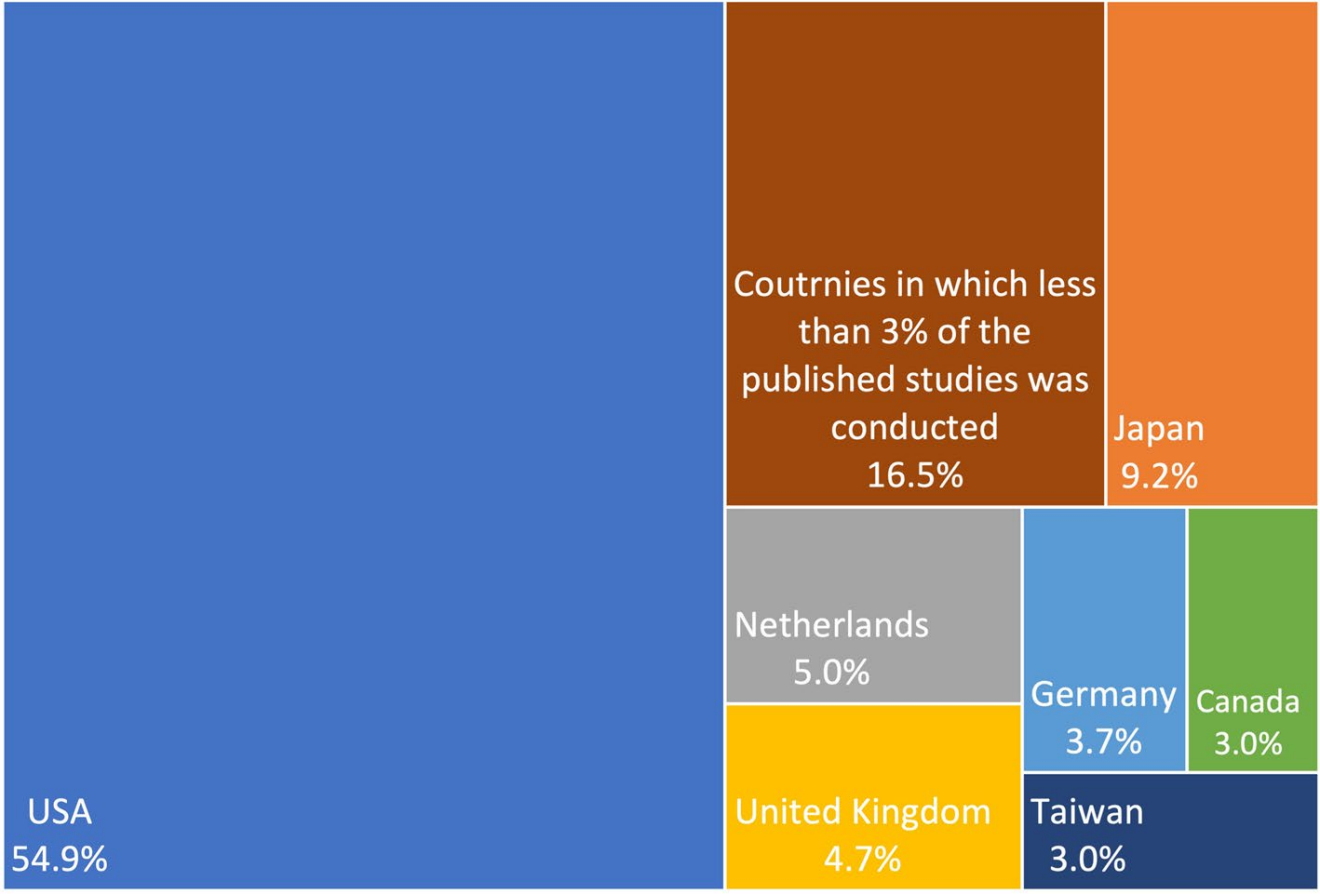
Proportions of studies performed in each country

Four hundred three publications from 188 different journals were included in the review. The studies were performed in 20 different countries, with more than half performed in the United States (54.9%; *n* = 221).The countries in which more than 3% of the studies were performed are represented in Fig. 2. Only 1 to 3% of the included studies were realised in Australia, Sweden, Finland, France, Korea, Spain, and this rate is lower than 1% for Italy, Belgium, Norway, South Korea, Brazil, Italia and Switzerland.

There were very few multinational studies (2.3%; *n* = 9).

The number of studies increased over time: a total of 24 papers were published during the period 2009-2011, whereas over 50 per year were published in 2018, 2019, and 2020.

### Data sources

One hundred ninety-six different databases were used to study the volume-outcome relationship. More than half of these (54.75%; *n* = 221) were administrative databases, as defined by Levac et al. [31]. Nearly a third of them were patient or disease registers (29.25%; *n* = 118), followed by claims databases (11.00%; *n* = 44), health surveys (2.25%; *n* = 9), and clinical trials data (1.75%; *n* = 7). Less than 1% of the included studies were based on data extracted from electronic health records.

### The surgical disciplines and procedures investigated

Among the 403 studies reviewed, the most represented surgical discipline was visceral and digestive tract surgery (37.75%; *n* = 152), followed by thoracic and cardiovascular surgery (11.25%; *n* = 45), urology (10.0%; *n* = 40), orthopaedic surgery (9.50%; *n* = 38), vascular surgery (8.0%; *n* = 32) and paediatric surgery (5.5%; *n* = 22). Other specialties were explored in less than 5% of the studies.

Ninety distinct types of surgical activity were explored. Almost half of the studies concerned oncological indications (191, 47.5%). More than 5% of the publications studied pancreatic surgery (5.24%; *n* = 21) followed by gastrectomy (3.74%;*n* = 15), esophagectomy (3.74%; *n* = 15), aortic and mitral valve surgery (3.74%; *n* = 15), rectal surgery (3.74%; *n* = 15), hip surgery (3.74%; *n* = 15), lung surgery (3.74%; *n* = 15), abdominal aortic aneurysms (3.49%; *n* = 14), and cystectomy (3.49%; *n* = 14). Other types of surgical activity were found in less than 3% of the studies.

In order to identify the patient populations undergoing surgery, 73.5% (*n* = 296) of the publications used a version of the ICD. The majority of the publications that did not use the ICD (65.1%) were based on patient or disease registers.

### Outcomes and hospital volume

Hospital volume was expressed as a categorical variable only in 80.2% of the publications, as a continuous variable only in 4.3%, and as a continuous variable and a categorical variable in 15.5%.

Among the studies of volume as a categorical variable, nearly half (49.2%) used quantiles. The other studies used literature definitions (19.8%), statistically defined cut-offs (5.8%), or other methods (18.5%). 6.6% of the studies assessed the volume as a categorical value in two or more ways.

Most of the included studies had more than one outcome. Mortality was the most frequently explored outcome (79.9%; *n* = 321), followed by length of stay (32.0%; *n* = 129), hospital readmission (16.6%; *n* = 67), and cost (16.1%; *n* = 65). 61.0% (*n* = 246) of the studies explored an outcome other than the four just mentioned. The most frequent of these were complications rates (32.1%; *n* = 79), followed by failure-to-rescue (death after a major complication) (7.0%; *n* = 17), specific oncological issues (5.5%; *n* = 17), morbidity (3.5%; *n* = 9), and discharge status (2.7%; *n* = 7).

All 61 outcome variables are listed in Table 5. They were grouped into nine families: length of stay, mortality, readmission, oncological issues, cost, characteristics of the hospital stay, quality indicators, surgical complications, and medical complications.

**Table 5:** Outcomes explored by the reviewed studies

Mortality	Length of stay	Hospital readmission	Cost	Surgical complications	General complications	Oncological issues	Characteristics of the hospital stay (continuous)	Quality indicators
Death occurring during a pre-defined time period (1, 7, 30, 60, 90 or 180 days, 1, 2, 3 or 5 years)	Continuous length of stay (from admission to discharge)	Readmission for any cause during a pre-defined time period (2 weeks, 30 days, 6 weeks, 90 days, or 1, 2, or 3 years)	Costs billed by the hospital, excluding physicians’ fees	Severity of the complication: Clavien-Dindo grade III or higher	At least one complication within a specific time period (in-hospital stay, 30 or 90 days, 75th percentile of the length of stay) or the complication rate	successful surgery: positive surgical margins (circumferential), number of resected lymph nodes, complete resection or not, restorative surgery performed or not.	Being in the intensive care unit (ICU) for longer than a specific time (1 or 2 days) or requiring organ support (mechanical ventilation or dialysis)	Patient safety indicators
Failure-to-rescue rates: death after a major complication within a pre-defined period of time (30 days, 90-days, in-hospital)	Continuous post-surgical length of stay (from surgery to discharge)	Readmission for a specific cause (aseptic revision, rescue surgery as a full procedure after a partial one, implant revision)	Sum of all costs except the cost of initial surgery (considered to be similar across all centres)	Abscess, haemorrhage, or anastomotic leakage	Major complications, defined as the need for surgical intervention or organ supply within a specific time period (in-hospital, 14 days)	Recurrence of cancer: local recurrence, distant metastases, and vital status	Routine discharge versus nonroutine discharge (i.e. to rehabilitation, home, or a care home)	Surgical reconstruction rate
Ratio between observed and expected (O/E) deaths	Continuous length of uninterrupted institutional care	A composite of readmission to an acute care hospital for any cause and all-cause death within a specific time period	Excess costs, defined as those above a defined cut-off (median, 75th percentile, etc.)	Surgical site bleeding, need for transfusion, or transfusion volume	Kidney failure or urinary tract infection	Risk ratios for positive margins	Time to surgery/transplantation or the door-to-balloon time	Proportion of pneumectomies
Risk-adjusted mortality: the O/E ratio multiplied by the overall mortality rate for the cohort	Prolonged length of stay: longer than a cut-off (insurance period, median value, 75th percentile, 90th percentile, etc.)	Risk-adjusted readmission rate, the O/E ratio multiplied by the overall readmission rate for the cohort	Cost per day of treatment: the total cost divided by the length of stay	Perforation or laceration (bowel, oesophageal, ureteral, rectum, bladder, nerve, etc.)	One of the eight emergency general surgery complications	Time to recurrence or progression-free survival time	Duration of treatment with antibiotics	Amputation-free survival
Hospital-standardized mortality ratio		Unplanned readmission to the same hospital	Cost-per-episode of surgery, adjusted for wage index	Surgical site infection, wound infection	Sepsis, septicaemia, shock, or prosthesis or implant infection		Travel time or distance to hospital	Sphincter preservation
Disease-related death (cancer, surgery, or sepsis related)		Readmission rate for each subspecialty or diagnosis	Cost-per-episode of surgery, adjusted for inflation	Major amputation (lower limb, organ)	Pulmonary embolism, deep venous thrombosis		Number of hours requiring organ support	Survival of thumb replantation
Time-to-death, as the time interval between surgery and death		Reoperation during the initial hospital stay			Neurological or cerebrovascular complications		Duration of surgery	
Post-discharge mortality					Need for a permanent pacemaker		Duration of anaesthesia	
In-hospital mortality					Respiratory failure or arrest			
					Cardiac arrest or myocardial infarction			

NB: the outcomes are not listed in a particular order

We have not reported the proportions for each outcome because many studies used several of these (e.g. 30-day mortality and in-hospital mortality).

### Covariates included in the model, and assessment of the initial severity

The 72 types of covariates used at least once for adjusting statistical models (listed in Table 6) were grouped into the following eight families, using an inductive approach: the patient’s characteristics, the hospital’s characteristics, clinical conditions, severity assessment, details of the disease, details of the surgery, details of the hospital stay, and post-operative events. Twenty five publications did not take into account any confounders when analyzing the hospital volume-outcome relationship. Five of the 25 studies (20%) did not find a significant hospital volume-outcome relationship. In contrast, only 12.6% of the studies that took account of potential confounders did not find this relationship.

**Table 6:** covariates used to adjust statistical models in at least one study

Patient characteristics	Hospital characteristics	Clinical conditions	Severity	Details of the disease	Details of the surgery	Details of the hospital stay	Post-operative events
Age (patient, donor, recipient, gestational age)	Hospital size (number of beds)	Laboratory results (albumin, anaemia, hyperlipidaemia, natremia, and creatinine)	The All Patient Refined Diagnosis Related Group score	Time to treatment (door-to-balloon time, waiting time, etc.)	Type of procedure and approach (laparoscopic, open, percutaneous, minimally invasive, etc.)	Admission source (nursing facility, home, transfer within hospital, or ambulance)	Adverse effects
Substance abuse/consumption (alcohol, illicit drugs, tobacco, etc.)	Hospital ownership or type (private or public sector)	Signs of shock on admission (heart rate, hypotension, Glasgow Coma Scale, Japan Coma Scale, hemodynamic instability, ECG)	The Charlson-Comorbidity Index [32]	Tumour characteristics (site, shape, size, stage, grade, differentiation status, histological subgroup, clinical-TNM, pre-therapeutic-TNM, extent, metastasis, distance from critical organs)	Concomitant procedures (additional resections, lymphadenectomy, and valve surgery)	Distance (from home, from previous facility, to the closest high-volume hospital, total distance travelled, etc.)	Resection status (complete resection of positive margins)
Sex or gender	Hospital teaching status or level (trauma centre, centre of excellence, reference centre, etc.)	Heart disease (atrial fibrillation, LVEF trend, NYHA class [33])	The Elixhauser score [34]	Injury severity and mechanism, trauma site	Details of anaesthesia (substance used, duration, and local or general)	Cost (total or out-of-pocket)	Complications
Language	Hospital location (urban, rural, city, state, country, region)	Lung disease (chronic obstructive pulmonary disease, asthma, chronic lung conditions)	The American Society of Anesthesiologists score (ASA) [35]	Multiple and/or synchronous tumours	Treatment modality (resection, laser, stenting, bariatric procedure, etc.)	Admission status (scheduled or emergency)	Post-operative radiotherapy or chemotherapy
Body mass index (or birthweight or obesity) or body surface area	Number of licensed practical nurses per occupied bed	Liver disease (cirrhosis or failure)	Self-reported comorbidities	Aneurysm characteristics (diameter and rupture)	Risk Adjustment for Congenital Heart Surgery [36]	Discharge type (transfer, recovery unit, nursing home, etc.)	
Geographic information (city, state, country, zip code, population, density)	Number of registered nurses per occupied bed	Renal disease (chronic or acute renal failure, creatinine, or dialysis)	The modified Kellgren-Lawrence score [37]	Specific scores: International Federation of Gynecology & Obstetrics [38], Furhman [39], Injury Severity Score [40], Kidney Donor Risk Index [41])	Surgical indication	Length of uninterrupted institutional care (LUIC)	
Economic status or income (self-reported, zip-code-based, quartiles)		Chronic diseases (diabetes and depression)	The Score for Neonatal Acute Physiology [42]	Lymph node status and lymphadenectomy	Surgeon’s specialty	Period of admission (the year and/or month, on a weekday vs. the weekend)	
Educational level (self-reported, parents’ level, zip-code-based)		Pre-operative medical treatment (neo-adjuvant chemotherapy or radiotherapy, immunosuppressive therapy, aspirin, beta blocker, biopsies, statins)	Pre-operative patient-reported outcome measures	Benign or malignant tumours	Time interval between diagnosis and surgery	Total length of stay	
Social security status (or primary payer)		Previous surgery (coronary surgery, aortic surgery, total hip/knee replacement, or carotid revascularization)		Diagnosis and/or aetiology	Intraoperative blood volume loss and/or transfusion requirement	Specific length of stay (LUIC, ICU, pre-surgery unit)	
Race or ethnicity		Previous or active cancer		Date of diagnosis	Oncological issues (concomitant examination and result, microscopic or macroscopic negative margins)	Ambulatory status	
		Family history of disease		Number of diagnoses		ICU stay	
				Primary tumour site			

*ECG* Electrocardiography, *LVEF* left ventricular ejection fraction, *NYHA* New York Heart Association, *TNM* tumour nodule metastasis classification, *ICU* intensive care unit, *LUIC* length of uninterrupted institutional care

### Statistically significant, positive volume-outcome relationships

A statistically significant relationship between hospital volume and outcome was found in 86.6% (*n* = 349) of the reviewed studies. Regardless of the volume modality, the type of outcome and the covariate(s) included in the model, 86.2% (*n* = 347) of the studies that assessed mortality found a significant relationship. Depending on the way that the volume was assessed, either a greater hospital volume was significantly associated with a lower mortality rate or a group of hospitals with a higher volume had a lower mortality rate that a group with a lower volume. Furthermore, volume was significantly related the length of stay (in 89.1% of the studies; *n* = 359), cost (89.1%; *n* = 359), and hospital readmission (79.1%; *n* = 319). A hospital volume-outcome relationship was also found in 87.3% (*n* = 352) of the studies that explored at least one outcome other than those just listed.

This relationship was found only in 66.7% (*n* = 269) of the studies performed in Korea, with values of 70.0% (*n* = 282) in Australia, 73.7% (*n* = 295) in the Netherlands, and 75% (*n* = 302) in Canada. For all other countries, the proportion of studies having found a statistically significant volume-outcome relationship was above 85%.

**Fig. 3:**
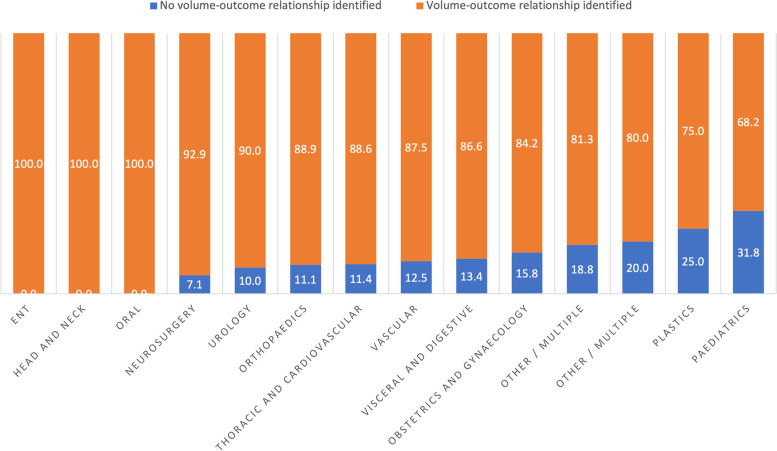
The percentage of studies having found a significant volume-outcome relationship, as a function of the discipline of surgery assessed

The proportion of studies having found a statistically significant, positive volume-outcome relationship was similar for cancer indications (88%) and other indications (85%). The proportion was lower for paediatrics (68.2%) and plastic surgery (75.0%) but greater than 80% for other specialties (Fig. 3). A volume-outcome relationship was not evidenced for five types of surgery: benign prostate hyperplasia, cholangiocarcinoma, intra-arterial stroke treatment, intracranial aneurysms, and necrotizing enterocolitis. Four types of surgery (appendicectomy, colorectal resection, infantile hypertrophic pyloric stenosis or pancreas transplantation) featured a volume-outcome relationship in less than 50% of the studies, and 6 types (liver transplantation, hysterectomy for cancer, congenital diaphragmatic hernia, nephrectomy, total joint arthroplasty, and abdominal aortic aneurysm) featured a volume-outcome relationship in between 50 and 75% of the studies (Supplementary Figure 1).

## Discussion

The objective of the present scoping review of the literature was to assess the ways in which the volume-outcome relationship was studied. Our main findings highlighted the diversity of the types of surgery, the types of outcome explored, and the method for exploring the volume-outcome relationship. The 403 studies included in the review variously assessed 90 types of surgery, 61 types of outcome, and 72 potential confounders.

Most of the studies (87.5%) of the volume-outcome relationship had been performed in Western countries (as defined by Huntington [43]). More than half of all the included studies were based on administrative databases (54.8%), even though the latter do not always describe all the patients treated in a given centre. In fact, some administrative databases only describe patients with social security coverage or other types of health insurance, and some (particularly in the USA) even describe only patients covered by a particular private healthcare provider. A proportion of the patient population specifically concerned by the volume-outcome issue might therefore have been excluded from these studies. In countries with low success rates, it would be interesting to look at why quality varied. The high proportion of Western countries may limit the degree to which the studies’ data can be extrapolated.

Nearly 50% of the studies assessed cancer surgery (47.5%), and a third assessed visceral or digestive tract surgery (37.8%). This distribution might not reflect actual levels of activity. By way of an example, only 8.1% of hospital stays for surgery in France in 2019 were for an oncological indication (vs. 47.5% in the present review). The corresponding values are 27.2% for orthopaedic surgery (9% in the review), 17.9% for ophthalmology (less than 5% here), 13.1% for digestive tract or visceral study (37.8% here), 9.1% for urology (10% here), and 5.5% for cardiovascular surgery (11.3% here); hence, the proportions found here do not match the activity data [44, 45]. Even when comparing our review’s results with the activity in the US reported by Stanford HealthCare in the United States in 2009, only 12.7% of operations concerned the digestive tract (37.8% here), with 2.3% for the urinary tract (10% here) and 15.2% for the cardiovascular system (11.3% here) [46]. Studies that found a positive volume-outcome relationship for rare, complex, specific types of surgery must be interpreted with caution, since they may not reflect surgical activity in general.

Although 86.6% of the reviewed studies found a statistically significant volume-outcome relationship, the results differed from one type of surgery to another. For example, a significant relationship was found in 70% of the studies of paediatric surgery and not at all for five specific types of surgery (benign prostate hyperplasia, cholangiocarcinoma, intra-arterial stroke treatment, intracranial aneurysms, and necrotizing enterocolitis).

Our review highlighted a high degree of diversity among the outcomes measured and the covariates included in statistical analyses. Even though almost 80% of the studies investigated mortality as one of their outcomes, the way it was assessed modified the end results. For example, some studies looked at 5-year mortality among a population of elderly patients in which life expectancy can be a major source of bias, whereas other looked at 1-day mortality. Cost (explored in 16.1% of the studies) always has a particular context and depends on the country in which it is studied. Indeed, the share of a given cost paid by the patient may differ markedly in the USA vs. France. Moreover, patient outcomes may be interlinked because nursing facilities in some countries (but not in others) have incentives to hospitalize residents [47].

This heterogeneity can be viewed as both a strength and a limitation, and a few studies have shown the results depend on the variable or analytical method used [13, 48]. In 2015, Yu et al. showed that categorization of volume as either a continuous variable, in quartiles or as k-means yielded different relationships with the outcome [14]. In 2018, Bernard et al. reported that four different regression models gave significantly different results for the same datasets [49].

Covariates also have a major impact on the assessment of the volume-outcome relationship. A recent study of cholangiocarcinoma resection showed that the relationship was no longer significant after adjustment for the distance travelled [50].

Volume may not be the only issue to be considered. For example, Mukhtar et al. compare high-activity years with low/medium-activity years in a San Francisco hospital year over a 15-year period; neither the complication rate nor the mortality rate depended on the surgical volume [51]. These results are suggestive of a learning curve effect. Indeed, centres that increased their volume year-on-year sometimes had better outcomes than centres with absolute volumes that were higher but decreased year-on-year [52, 53].

The study populations in high-volume centres and low-volume centres are probably not the same, and thus should be taken into account in the analytical model. Indeed, Liu et al.’s 2017 study of cancer surgery showed that patient attendance at low-volume centres was associated with a shorter travelling distance, residence in a rural area, and the absence of neoadjuvant therapy but not with the severity of their disease [54]. In 2017, Gani et al. showed that ethnic minorities, elderly patients, and patients with many comorbidities may have more difficulty accessing high-volume centres, which increases inequalities in access to care [15].

Even though the great majority of studies (in almost all surgical fields and all countries) found a volume-outcome relationship, those that explored centralization showed that having only high-volume centre had adverse effects and might not improve patient outcomes. Stitzenberg et al. reported that a marked increase in travelling distance observed after the centralization of pancreatic surgery posed a significant obstacle to accessing quality care [55] and increased inequalities in care access for specific populations - mainly in rural states [56]. Dimick et al. even suggested that given the size of the USA and the numbers of some types of surgery, nationwide local access to a high-volume facility is impossible [57].

The great variety of outcomes and covariates used to assess the hospital volume-outcome relationship, the high predominance of studies in Western countries, and the over-representation of oncological, visceral and digestive tract surgery may limit the generalizability of the studies’ results. Given the many different ways in which this relationship has been explored, policymakers should be very careful when using the conclusions of specific studies to modifying healthcare facility maps.

This review suffered from several limitations. Firstly, the study’s design as a scoping review prevented us from evaluating the methodological quality of each study included. Secondly, our predefined categories may not have been precise enough to analyse each type of study. Indeed, the database categories, the types of surgery and the statistical methods could have been more precise. However, with a view to overcoming this limitation, the extraction grid was first tested on 10 studies. Thirdly, our literature search was limited to two electronic databases (PUBMED and Scopus) and the search terms selected may not be exhaustive. Hence, other relevant publications in other databases, or presenting none of the included keywords would have been missed [58]. Fourthly, our review was limited to the scientific literature and thus did not cover the pricing data used by policy makers to take decisions about healthcare facility mapping. Lastly, we reviewed the hospital volume-outcome relationship for surgery in general. Hence, our results may be relevant from the hospital perspective rather than that of individual surgeons.

The present review is the first to provide an exhaustive overview of how volume-outcome relationship has been explored and how relevant criteria can be selected as a function of a study’s objective. Results showed that even if most of the study showed a significant volume-outcome relationship, every feature of the analysis provide a different information. In consequence, before using such results to adapt a health facility mapping, policy-makers should perform a specific study on the surgery and territory of interest. In order to help them with such analysis, this review tries to provide a set of tools for investigating the volume-outcome relationship that can be adapted depending on the desired goal.

## Supplementary Information

**Additional file 1 **: **Supplementary Figure 1**. The percentage of studies in which a significant volume-outcome relationship, as a function of the type of surgery assessed.

**Additional file 2 **: **Supplementary Table 1.** Extraction data form.

**Additional file 3.** Reference list of the studies included in the review.

## Data Availability

The datasets used and/or analysed during the current study available from the corresponding author on reasonable request.
