# Supplementary figures and images for: Assessing the hospital volume-outcome relationship in surgery: a scoping review
Source: BMC Med Res Methodol. 2021 Oct 9;21:204. doi: 10.1186/s12874-021-01396-6 (PMC8502281; doi:10.1186/s12874-021-01396-6)

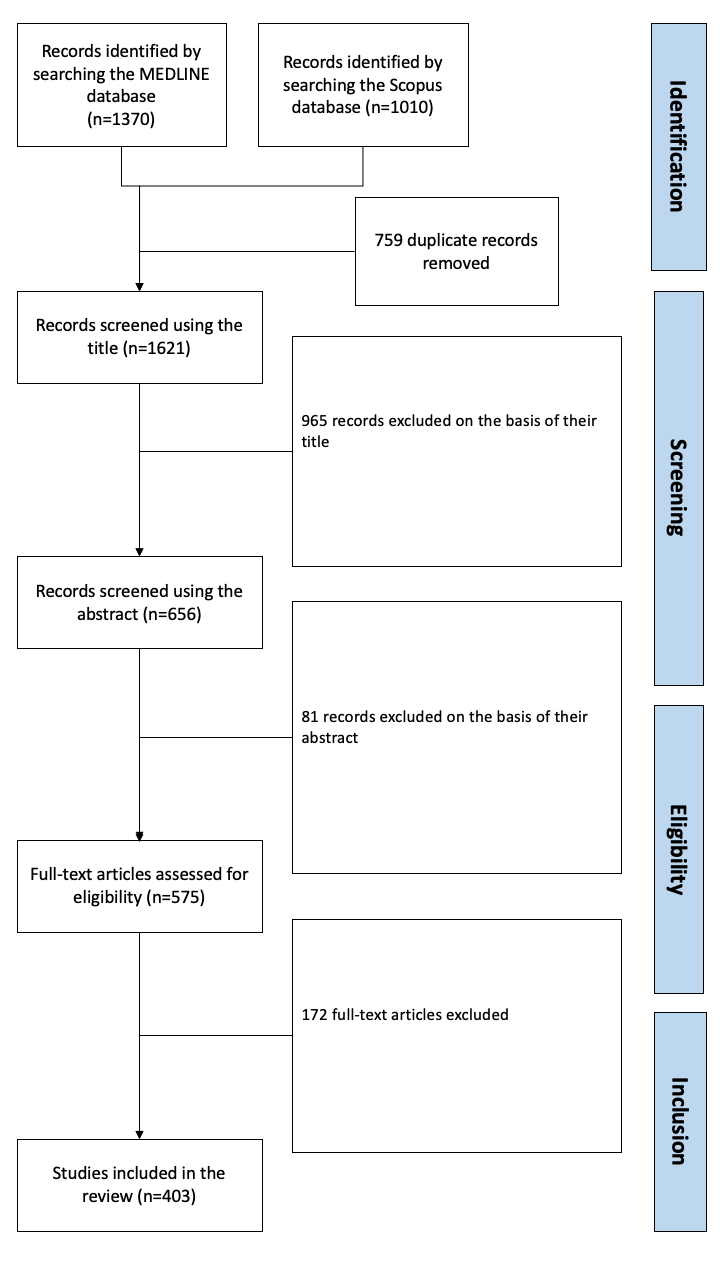

Supplement: Supplementary file 1 — Additional file 1 : Supplementary Figure 1. The percentage of studies in which a significant volume-outcome relationship, as a function of the type of surgery assessed. [file 12874_2021_1396_MOESM1_ESM.png]
