# Supplementary material for: Assessing the hospital volume-outcome relationship in surgery: a scoping review
Source: BMC Med Res Methodol. 2021 Oct 9;21:204. doi: 10.1186/s12874-021-01396-6 (PMC8502281; doi:10.1186/s12874-021-01396-6)
Supplement: Supplementary file 2 — Additional file 2 : Supplementary Table 1. Extraction data form. [file 12874_2021_1396_MOESM2_ESM.docx]

| **Supplementary table 1 – Extraction data form** | |
| --- | --- |
| Title | ___________________________________ |
| Author | ___________________________________ |
| Year of publication | ___________________________________ |
| Research design | ___________________________________ |
| Research objectives | ___________________________________ |
| Type of sources | ❒ Published ❒ Grey literature |
| Specific information | ___________________________________ |
| 1. Database used | ___________________________________ |
|  | ❒ Electronic health record ❒ Administrative data  ❒ Claims data ❒ Patient or disease register  ❒ Health survey ❒ Clinical trials data |
| 2. Surgery studied | ___________________________________ |
| ❒ Head and Neck ❒ Oral ❒ Orthopaedics ❒ Paediatrics ❒ Plastics ❒ Vascular  ❒ Thoracic and cardiovascular ❒ Visceral and digestive ❒ Neurosurgery  ❒ ENT ❒ Obstetrics and gynaecology ❒ Ophthalmology ❒ Urology | |
| 3. Use of the International Classification of Disease | ❒ Yes ❒ No |
| 4. Inclusion and exclusion criteria | ___________________________________ |
| 5. Method to sort hospitals by volume | ❒ Continuous ❒ Categorical ❒ Both |
| If continuous or both, precise the method used: | ❒ Fractals ❒ Statistical cut-off ❒ Arbitrary ❒ Other |
| 6. Outcomes | ❒ Mortality ❒ Cost ❒ Length of stay  ❒ Readmission ❒ Other |
| If other, precise the interesting outcome studied | ___________________________________ |
| 7. Severity score | ❒ Charlson/Deyo ❒ Elixhauser ❒ Other |
| and covariates | ___________________________________ |
| 8. Statistical methods | ❒ One sample test ❒ Two independent sample test  ❒ Two correlated sample test ❒ More than two independent sample test ❒ More than two correlated sample test ❒ Correlation ❒ Two categorical variables ❒ Same categorical outcome on matched pairs  ❒ Linear multiple regression ❒ Other |
| If other, precise | ___________________________________ |
| 9. Results’ presentation | ❒ Graph(s) ❒ Table(s) ❒ Both |
| If graph or both, precise its typology | ❒ Line graph ❒ Bar graph ❒ Scatter gram ❒ Histogram ❒ Pie chart ❒ Boxplot ❒ Other |
| 10. Conclusion: positive volume-outcome relationship | ❒ Yes ❒ No |
