# Supplementary material for: Assessing the hospital volume-outcome relationship in surgery: a scoping review
Source: BMC Med Res Methodol. 2021 Oct 9;21:204. doi: 10.1186/s12874-021-01396-6 (PMC8502281; doi:10.1186/s12874-021-01396-6)
Supplement: Supplementary file 3 — Additional file 3. Reference list of the studies included in the review. [file 12874_2021_1396_MOESM3_ESM.docx]

**Supplementary material – References of all the studies’ included**

1. Adam MA, Moris D, Behrens S, Nussbaum DP, Jawitz O, Turner M, et al. Hospital Volume Threshold for the Treatment of Retroperitoneal Sarcoma. Anticancer Res. 2019;39(4):2007–14.

2. Adam MA, Thomas S, Youngwirth L, Pappas T, Roman SA, Sosa JA. Defining a Hospital Volume Threshold for Minimally Invasive Pancreaticoduodenectomy in the United States. JAMA Surg. 2017 Apr 1;152(4):336.

3. Ahola R, Sand J, Laukkarinen J. Pancreatic resections are not only safest but also most cost-effective when performed in a high-volume centre: A Finnish register study. Pancreatology. 2019 Jul;19(5):769–74.

4. Alali AS, Gomez D, McCredie V, Mainprize TG, Nathens AB. Understanding Hospital Volume–Outcome Relationship in Severe Traumatic Brain Injury. Neurosurgery. 2017 Apr 1;80(4):534–42.

5. Albornoz CR, Cordeiro PG, Hishon L, Mehrara BJ, Pusic AL, McCarthy CM, et al. A Nationwide Analysis of the Relationship between Hospital Volume and Outcome for Autologous Breast Reconstruction: Plastic and Reconstructive Surgery. 2013 Aug;132(2):192e–200e.

6. Alsfasser G, Leicht H, Günster C, Rau BM, Schillinger G, Klar E. Volume-outcome relationship in pancreatic surgery. Br J Surg. 2016 Jan;103(1):136–43.

7. Alsfasser G, Kittner J, Eisold S, Klar E. Volume-outcome relationship in pancreatic surgery: The situation in Germany. Surgery. 2012 Sep;152(3):S50–5.

8. Altieri MS, Yang J, Groves D, Yin D, Cagino K, Talamini M, et al. Academic status does not affect outcome following complex hepato-pancreato-biliary procedures. Surg Endosc. 2018 May;32(5):2355–64.

9. Amini N, Spolverato G, Kim Y, Pawlik TM. Trends in Hospital Volume and Failure to Rescue for Pancreatic Surgery. J Gastrointest Surg. 2015 Sep;19(9):1581–92.

10. Anderson O, Ni Z, Møller H, Coupland VH, Davies EA, Allum WH, et al. Hospital volume and survival in oesophagectomy and gastrectomy for cancer. Eur J Cancer. 2011 Nov;47(16):2408–14.

11. Andersson RE. Short and Long-Term Mortality After Appendectomy in Sweden 1987 to 2006. Influence of Appendectomy Diagnosis, Sex, Age, Co-morbidity, Surgical Method, Hospital Volume, and Time Period. A National Population-Based Cohort Study. World J Surg. 2013 May;37(5):974–81.

12. Ando T, Adegbala O, Villablanca PA, Shokr M, Akintoye E, Briasoulis A, et al. Failure to Rescue, Hospital Volume, and In-Hospital Mortality After Transcatheter Aortic Valve Implantation. The American Journal of Cardiology. 2018 Sep;122(5):828–32.

13. Andresen NS, Gourin CG, Stewart CM, Sun DQ. Hospital volume and failure to rescue after vestibular schwannoma resection. The Laryngoscope. 2019 Jul 3;lary.28174.

14. Antila A, Ahola R, Sand J, Laukkarinen J. Management of postoperative complications may favour the centralization of distal pancreatectomies. Nationwide data on pancreatic distal resections in Finland 2012–2014. Pancreatology. 2019 Jan;19(1):26–30.

15. Aquina CT, Probst CP, Becerra AZ, Iannuzzi JC, Kelly KN, Hensley BJ, et al. High volume improves outcomes: The argument for centralization of rectal cancer surgery. Surgery. 2016 Mar;159(3):736–48.

16. Arias-de la Torre J, Valderas JM, Evans JP, Martín V, Molina AJ, Muñoz L, et al. Differences in Risk of Revision and Mortality Between Total and Unicompartmental Knee Arthroplasty. The Influence of Hospital Volume. J Arthroplasty. 2019;34(5):865–71.

17. Arora S, Panaich SS, Patel N, Patel N, Lahewala S, Solanki S, et al. Impact of Hospital Volume on Outcomes of Lower Extremity Endovascular Interventions (Insights from the Nationwide Inpatient Sample [2006 to 2011]). The American Journal of Cardiology. 2015 Sep;116(5):791–800.

18. Arora S, Keeley J, Pucheril D, Menon M, Rogers CG. What is the hospital volume threshold to optimize inpatient complication rate after partial nephrectomy? Urologic Oncology: Seminars and Original Investigations. 2018 Jul;36(7):339.e17-339.e23.

19. Asano EF, Rasera I, Shiraga EC. Cross-sectional study of variables associated with length of stay and ICU need in open Roux-En-Y gastric bypass surgery for morbid obese patients: an exploratory analysis based on the Public Health System administrative database (Datasus) in Brazil. Obes Surg. 2012 Dec;22(12):1810–7.

20. Auerbach AD, Hilton JF, Maselli J, Pekow PS, Rothberg MB, Lindenauer PK. Shop for Quality or Volume? Volume, Quality, and Outcomes of Coronary Artery Bypass Surgery. :10.

21. Avritscher EBC, Cooksley CD, Rolston KV, Swint JM, Delclos GL, Franzini L, et al. Serious postoperative infections following resection of common solid tumors: outcomes, costs, and impact of hospital surgical volume. Support Care Cancer. 2014 Feb;22(2):527–35.

22. Axt JR, Murphy AJ, Arbogast PG, Lovvorn HN. Volume-outcome effects for children undergoing resection of renal malignancies. Journal of Surgical Research. 2012 Sep;177(1):e27–33.

23. Babadjouni R, Wen T, Donoho DA, Buchanan IA, Cen SY, Friedman RA, et al. Increased Hospital Surgical Volume Reduces Rate of 30- and 90-Day Readmission After Acoustic Neuroma Surgery. Neurosurgery. 2019 Mar 1;84(3):726–32.

24. Badawy M, Espehaug B, Indrekvam K, Engesæter LB, Havelin LI, Furnes O. Influence of Hospital Volume on Revision Rate After Total Knee Arthroplasty with Cement: The Journal of Bone & Joint Surgery. 2013 Sep;95(18):e131.

25. Badawy M, Fenstad AM, Bartz-Johannessen CA, Indrekvam K, Havelin LI, Robertsson O, et al. Hospital volume and the risk of revision in Oxford unicompartmental knee arthroplasty in the Nordic countries -an observational study of 14,496 cases. BMC Musculoskelet Disord. 2017 Sep 7;18(1):388.

26. Badheka AO, Patel NJ, Panaich SS, Patel SV, Jhamnani S, Singh V, et al. Effect of Hospital Volume on Outcomes of Transcatheter Aortic Valve Implantation. Am J Cardiol. 2015 Aug 15;116(4):587–94.

27. Baek J-H, Alrubaie A, Guzman EA, Choi SK, Anderson C, Mills S, et al. The association of hospital volume with rectal cancer surgery outcomes. Int J Colorectal Dis. 2013 Feb;28(2):191–6.

28. Bagaria SP, Neville M, Gray RJ, Gabriel E, Ashman JB, Attia S, et al. The Volume-Outcome Relationship in Retroperitoneal Soft Tissue Sarcoma: Evidence of Improved Short- and Long-Term Outcomes at High-Volume Institutions. Sarcoma. 2018 Jul 24;2018:1–10.

29. Balentine CJ, Naik AD, Robinson CN, Petersen NJ, Chen GJ, Berger DH, et al. Association of high-volume hospitals with greater likelihood of discharge to home following colorectal surgery. JAMA Surg. 2014 Mar;149(3):244–51.

30. Barbas AS, Dib MJ, Rege AS, Vikraman DS, Sudan DL, Knechtle SJ, et al. The Volume-outcome Relationship in Deceased Donor Kidney Transplantation and Implications for Regionalization: Annals of Surgery. 2018 Jun;267(6):1169–72.

31. Baré M, Cabrol J, Real J, Navarro G, Campo R, Pericay C, et al. In-hospital mortality after stomach cancer surgery in Spain and relationship with hospital volume of interventions. BMC Public Health. 2009 Dec;9(1):312.

32. Bateni SB, Olson JL, Hoch JS, Canter RJ, Bold RJ. Drivers of Cost for Pancreatic Surgery: It’s Not About Hospital Volume. Ann Surg Oncol. 2018 Dec;25(13):3804–11.

33. Beal EW, Mehta R, Hyer JM, Paredes A, Merath K, Dillhoff ME, et al. Association Between Travel Distance, Hospital Volume, and Outcomes Following Resection of Cholangiocarcinoma. J Gastrointest Surg. 2019 May;23(5):944–52.

34. Beal EW, Mehta R, Merath K, Tsilimigras DI, Hyer JM, Paredes A, et al. Outcomes After Resection of Hepatocellular Carcinoma: Intersection of Travel Distance and Hospital Volume. J Gastrointest Surg. 2019 Jul;23(7):1425–34.

35. Becher RD, DeWane MP, Sukumar N, Stolar MJ, Gill TM, Maung AA, et al. Hospital Volume and Operative Mortality for General Surgery Operations Performed Emergently in Adults: Journal of Craniofacial Surgery. 2019 Feb;1.

36. Becher RD, DeWane MP, Sukumar N, Stolar MJ, Gill TM, Becher RM, et al. Hospital Operative Volume and Quality Indication for General Surgery Operations Performed Emergently in Geriatric Patients. J Am Coll Surg. 2019 Jun;228(6):910–23.

37. Becker JH, Ezendam NPM, Boll D, van der Aa M, Pijnenborg JMA. Effects of surgical volumes on the survival of endometrial carcinoma. Gynecol Oncol. 2015 Nov;139(2):306–11.

38. Berg S, Cole AP, Fletcher SA, Pucheril D, Nabi J, Lipsitz SR, et al. Investigating the effect of treatment at high-volume hospitals on overall survival following cytoreductive nephrectomy. Urologic Oncology: Seminars and Original Investigations. 2018 Sep;36(9):400.e15-400.e22.

39. Bernard A, Cottenet J, Mariet A-S, Quantin C, Pagès P-B. Is an activity volume threshold really realistic for lung cancer resection? J Thorac Dis. 2018 Oct;10(10):5685–94.

40. Bhatt P, Patel A, Kumar V, Lekshminarayanan A, Patel V, Alapati S, et al. Impact of hospital volume on outcomes of percutaneous ASD/PFO closure in pediatric patients. World J Pediatr. 2018 Aug;14(4):364–72.

41. Bhatt P, Patel NJ, Patel A, Sonani R, Patel A, Panaich SS, et al. Impact of Hospital Volume on Outcomes of Endovascular Stenting for Adult Aortic Coarctation. The American Journal of Cardiology. 2015 Nov;116(9):1418–24.

42. Bianco V, Aranda‐Michel E, Sultan I, Gleason TG, Chu D, Navid F, et al. Inconsistent correlation between procedural volume and publicly reported outcomes in adult cardiac operations. J Card Surg. 2019 Nov;34(11):1194–203.

43. Blais MB, Rider SM, Sturgeon DJ, Blucher J, Zampini JM, Kang JD, et al. Establishing objective volume-outcome measures for anterior and posterior cervical spine fusion. Clin Neurol Neurosurg. 2017 Oct;161:65–9.

44. Bliss LA, Maguire LH, Chau Z, Yang CJ, Nagle DA, Chan AT, et al. Readmission After Resections of the Colon and Rectum: Predictors of a Costly and Common Outcome. Diseases of the Colon & Rectum. 2015 Dec;58(12):1164–73.

45. Borowski DW, Bradburn DM, Mills SJ, Bharathan B, Wilson RG, Ratcliffe AA, et al. Volume-outcome analysis of colorectal cancer-related outcomes. Br J Surg. 2010 May 26;97(9):1416–30.

46. Bos ACRK, van Erning FN, Elferink MAG, Rutten HJ, van Oijen MGH, de Wilt JHW, et al. No Difference in Overall Survival Between Hospital Volumes for Patients With Colorectal Cancer in The Netherlands: Diseases of the Colon & Rectum. 2016 Oct;59(10):943–52.

47. Bozic KJ, Maselli J, Pekow PS, Lindenauer PK, Vail TP, Auerbach AD. The Influence of Procedure Volumes and Standardization of Care on Quality and Efficiency in Total Joint Replacement Surgery: The Journal of Bone and Joint Surgery-American Volume. 2010 Nov;92(16):2643–52.

48. Brescia AA, Patel HJ, Likosky DS, Watt TMF, Wu X, Strobel RJ, et al. Volume-Outcome Relationships in Surgical and Endovascular Repair of Aortic Dissection. The Annals of Thoracic Surgery. 2019 Nov;108(5):1299–306.

49. Brown EG, Bateni SB, Burgess D, Li C-S, Bold RJ. Interhospital Variability in Quality Outcomes of Pancreatic Surgery. Journal of Surgical Research. 2019 Mar;235:453–8.

50. Brown JB, Rosengart MR, Kahn JM, Mohan D, Zuckerbraun BS, Billiar TR, et al. Impact of Volume Change Over Time on Trauma Mortality in the United States: Annals of Surgery. 2017 Jul;266(1):173–8.

51. Brown M, Lu Y, Chung KC, Mahmoudi E. Annual Hospital Volume and Success of Digital Replantation: Plastic and Reconstructive Surgery. 2017 Mar;139(3):672–80.

52. Brunaud L, Polazzi S, Lifante J-C, Pascal L, Nocca D, Duclos A. Health Care Institutions Volume Is Significantly Associated with Postoperative Outcomes in Bariatric Surgery. Obes Surg. 2018;28(4):923–31.

53. Bucher BT, Guth RM, Saito JM, Najaf T, Warner BW. Impact of Hospital Volume on In-Hospital Mortality of Infants Undergoing Repair of Congenital Diaphragmatic Hernia: Transactions of the . Meeting of the American Surgical Association. 2010;128:243–52.

54. Bucknor A, Chattha A, Wu W, Egeler S, Ultee K, Afshar S, et al. The Impact of Surgical Volume on Outcomes and Cost in Cleft Repair: A Kidsʼ Inpatient Database Analysis. Annals of Plastic Surgery. 2018 Apr;80:S174–7.

55. Calderwood MS, Kleinman K, Huang SS, Murphy MV, Yokoe DS, Platt R. Surgical Site Infections: Volume-Outcome Relationship and Year-to-Year Stability of Performance Rankings. Medical Care. 2017 Jan;55(1):79–85.

56. CAMBErlin C, VriJEnS F, GAuquiEr KD, DEVriESE S. Provider volume and short term complications after elective total hip replacement : An analysis of Belgian administrative data. 2011;77:10.

57. Chattha A, Muste J, Patel A. The impact of hospital volume on clinical and economic outcomes in ventral hernia repair: an analysis with national policy implications. Hernia. 2018 Oct;22(5):793–9.

58. Chattha A, Bucknor A, Curiel DA, Ultee KHJ, Afshar S, Lin SJ. Treatment of Craniosynostosis: The Impact of Hospital Surgical Volume on Cost, Resource Utilization, and Outcomes. Journal of Craniofacial Surgery. 2018 Jul;29(5):1233–6.

59. Cheung MC, Koniaris LG, Perez EA, Molina MA, Goodwin WJ, Salloum RM. Impact of Hospital Volume on Surgical Outcome for Head and Neck Cancer. Ann Surg Oncol. 2009 Apr;16(4):1001–9.

60. Chiu C-C, Wang J-J, Tsai T-C, Chu C-C, Shi H-Y. The Relationship between Volume and Outcome after Bariatric Surgery: A Nationwide Study in Taiwan. OBES SURG. 2012 Jul;22(7):1008–15.

61. Choi H, Yang S-Y, Cho H-S, Kim W, Park E-C, Han K-T. Mortality differences by surgical volume among patients with stomach cancer: a threshold for a favorable volume-outcome relationship. World J Surg Onc. 2017 Dec;15(1):134.

62. Claassen YHM, van Sandick JW, Hartgrink HH, Dikken JL, De Steur WO, van Grieken NCT, et al. Association between hospital volume and quality of gastric cancer surgery in the CRITICS trial. Br J Surg. 2018;105(6):728–35.

63. Clement RC, Strassle PD, Ostrum RF. Should All Orthopaedists Perform Hemiarthroplasty for Femoral Neck Fractures? A Volume–Outcome Analysis: Journal of Orthopaedic Trauma. 2018 Jul;32(7):354–60.

64. Cobb AN, Wong YM, Brownlee SA, Blanco BA, Ezure Y, Paddock HN, et al. Perioperative support, not volume, is necessary to optimize outcomes in surgical management of necrotizing enterocolitis. The American Journal of Surgery. 2017 Mar;213(3):502–6.

65. Couapel J-P, Bensalah K, Bernhard J-C, Pignot G, Zini L, Lang H, et al. Is there a volume–outcome relationship for partial nephrectomy? World J Urol. 2014 Oct;32(5):1323–9.

66. Coupland VH, Konfortion J, Jack RH, Allum W, Kocher HM, Riaz SP, et al. Resection rate, hospital procedure volume and survival in pancreatic cancer patients in England: Population-based study, 2005–2009. European Journal of Surgical Oncology (EJSO). 2016 Feb;42(2):190–6.

67. Coupland VH, Lagergren J, Lüchtenborg M, Jack RH, Allum W, Holmberg L, et al. Hospital volume, proportion resected and mortality from oesophageal and gastric cancer: a population-based study in England, 2004-2008. Gut. 2013 Jul;62(7):961–6.

68. Damhuis RA, Maat AP, Plaisier PW. Performance indicators for lung cancer surgery in the Netherlands. Eur J Cardiothorac Surg. 2015 May;47(5):897–904.

69. David EA, Cooke DT, Chen Y, Perry A, Canter RJ, Cress R. Surgery in high-volume hospitals not commission on cancer accreditation leads to increased cancer-specific survival for early-stage lung cancer. The American Journal of Surgery. 2015 Oct;210(4):643–7.

70. de Biasi AR, Paul S, Nasar A, Girardi LN, Salemi A. National Analysis of Short-Term Outcomes and Volume-Outcome Relationships for Transcatheter Aortic Valve Replacement in the Era of Commercialization. Cardiology. 2016;133(1):58–68.

71. De la Garza-Ramos R, Abt NB, Kerezoudis P, Krauss W, Bydon M. Provider volume and short-term outcomes following surgery for spinal metastases. Journal of Clinical Neuroscience. 2016 Feb;24:43–6.

72. de Wilde RF, Besselink MGH, van der Tweel I, de Hingh IHJT, van Eijck CHJ, Dejong CHC, et al. Impact of nationwide centralization of pancreaticoduodenectomy on hospital mortality. Br J Surg. 2012 Mar;99(3):404–10.

73. Derogar M, Blomberg J, Sadr‐Azodi O. Hospital teaching status and volume related to mortality after pancreatic cancer surgery in a national cohort. BJS (British Journal of Surgery). 2015;102(5):548–57.

74. Dikken JL, van Sandick JW, Allum WH, Johansson J, Jensen LS, Putter H, et al. Differences in outcomes of oesophageal and gastric cancer surgery across Europe. Br J Surg. 2013 Jan;100(1):83–94.

75. Dikken JL, Dassen AE, Lemmens VEP, Putter H, Krijnen P, van der Geest L, et al. Effect of hospital volume on postoperative mortality and survival after oesophageal and gastric cancer surgery in the Netherlands between 1989 and 2009. Eur J Cancer. 2012 May;48(7):1004–13.

76. Dimick JB, Osborne NH, Nicholas L, Birkmeyer JD. Identifying High-Quality Bariatric Surgery Centers: Hospital Volume or Risk-Adjusted Outcomes? Journal of the American College of Surgeons. 2009 Dec;209(6):702–6.

77. Dua A, Furlough CL, Ray H, Sharma S, Upchurch GR, Desai SS. The effect of hospital factors on mortality rates after abdominal aortic aneurysm repair. Journal of Vascular Surgery. 2014 Dec;60(6):1446–51.

78. Dubrovsky G, Sacks GD, Friedlander S, Lee S. Understanding the relationship between hospital volume and patient outcomes for infants with gastroschisis. Journal of Pediatric Surgery. 2017 Dec;52(12):1977–80.

79. Dy CJ, Bozic KJ, Pan TJ, Wright TM, Padgett DE, Lyman S. Risk Factors for Early Revision After Total Hip Arthroplasty: Early Revision After THA: Risk Factors. Arthritis Care & Research. 2014 Jun;66(6):907–15.

80. El Amrani M, Clement G, Lenne X, Farges O, Delpero J-R, Theis D, et al. Failure-to-rescue in Patients Undergoing Pancreatectomy: Is Hospital Volume a Standard for Quality Improvement Programs? Nationwide Analysis of 12,333 Patients. Annals of Surgery. 2018 Nov;268(5):799–807.

81. El Amrani M, Clement G, Lenne X, Rogosnitzky M, Theis D, Pruvot F-R, et al. The Impact of Hospital Volume and Charlson Score on Postoperative Mortality of Proctectomy for Rectal Cancer: A Nationwide Study of 45,569 Patients. Annals of Surgery. 2018 Nov;268(5):854–60.

82. Elkassabany NM, Passarella M, Mehta S, Liu J, Neuman MD. Hospital characteristics, inpatient processes of care, and readmissions among older adults with hip fractures. J Am Geriatr Soc. 2016 Aug;64(8):1656–61.

83. Encinosa WE, Bernard DM, Du D, Steiner CA. Recent Improvements in Bariatric Surgery Outcomes: Medical Care. 2009 May;47(5):531–5.

84. Endo H, Fushimi K, Otomo Y. Volume–outcome relationship in severe operative trauma surgery: A retrospective cohort study using a Japanese nationwide administrative database. Surgery. 2019 Dec;166(6):1105–10.

85. Englot DJ, Ouyang D, Wang DD, Rolston JD, Garcia PA, Chang EF. Relationship between hospital surgical volume, lobectomy rates, and adverse perioperative events at US epilepsy centers. JNS. 2013 Jan;118(1):169–74.

86. Eskander A, Irish J, Groome PA, Freeman J, Gullane P, Gilbert R, et al. Volume-outcome relationship for head and neck cancer surgery in a universal health care system: Universal Healthcare Outcomes in HNSCC. The Laryngoscope. 2014 Sep;124(9):2081–8.

87. Feinglass J, Sohn M-W, Rodriguez H, Martin GJ, Pearce WH. Perioperative outcomes and amputation-free survival after lower extremity bypass surgery in California hospitals, 1996-1999, with follow-up through 2004. Journal of Vascular Surgery. 2009 Oct;50(4):776-783.e1.

88. Finks JF, Birkmeyer JD. Trends in Hospital Volume and Operative Mortality for High-Risk Surgery. n engl j med. 2011;10.

89. Fisher JH, Shapera S, To T, Marras TK, Gershon A, Dell S. Procedure volume and mortality after surgical lung biopsy in interstitial lung disease. Eur Respir J. 2019 Feb;53(2):1801164.

90. Forte ML, Virnig BA, Swiontkowski MF, Bhandari M, Feldman R, Eberly LE, et al. Ninety-Day Mortality After Intertrochanteric Hip Fracture: Does Provider Volume Matter?: The Journal of Bone and Joint Surgery-American Volume. 2010 Apr;92(4):799–806.

91. Frankel WC, Navarro SM, Haeberle HS, Ramanathan D, Ramkumar PN. Optimizing the Volume–Value Relationship in Laminectomy: An Evidence-Based Analysis of Outcomes and Economies of Scale. SPINE. 2019 May;44(9):659–69.

92. Freifeld Y, Woldu SL, Singla N, Clinton T, Bagrodia A, Hutchinson R, et al. Impact of Hospital Case Volume on Outcomes Following Radical Nephrectomy and Inferior Vena Cava Thrombectomy. European Urology Oncology. 2019 Nov;2(6):691–8.

93. Frisch NB, Courtney PM, Darrith B, Della Valle CJ. Do higher-volume hospitals provide better value in revision hip and knee arthroplasty? The Bone & Joint Journal. 2017 Dec;99-B(12):1611–7.

94. Fuchs HF, Harnsberger CR, Broderick RC, Chang DC, Sandler BJ, Jacobsen GR, et al. Mortality after esophagectomy is heavily impacted by center volume: retrospective analysis of the Nationwide Inpatient Sample. Surg Endosc. 2017 Jun;31(6):2491–7.

95. Fujita H, Ozawa S, Kuwano H, Ueda Y, Hattori S, Yanagawa T, et al. Esophagectomy for cancer: clinical concerns support centralizing operations within the larger hospitals. Dis Esophagus. 2010 Feb;23(2):145–52.

96. Fumagalli U, Bersani M, Russo A, Melis A, de Pascale S, Rosati R. Volume and outcomes after esophageal cancer surgery: the experience of the Region of Lombardy—Italy. Updates Surg. 2013 Dec;65(4):271–5.

97. Furuya-Kanamori L, Doi SAR, Smith PN, Bagheri N, Clements ACA, Sedrakyan A. Hospital effect on infections after four major surgical procedures: outlier and volume–outcome analysis using all-inclusive state data. Journal of Hospital Infection. 2017 Oct;97(2):115–21.

98. Gani F, Azoulay D, Pawlik TM. Evaluating Trends in the Volume-Outcomes Relationship Following Liver Surgery: Does Regionalization Benefit All Patients the Same? J Gastrointest Surg. 2017;21(3):463–71.

99. Gani F, Johnston FM, Nelson-Williams H, Cerullo M, Dillhoff ME, Schmidt CR, et al. Hospital Volume and the Costs Associated with Surgery for Pancreatic Cancer. J Gastrointest Surg. 2017 Sep;21(9):1411–9.

100. Geisbüsch S, Kuehnl A, Salvermoser M, Reutersberg B, Trenner M, Eckstein H-H. Editor’s Choice - Hospital Incidence, Treatment, and In Hospital Mortality Following Open and Endovascular Surgery for Thoraco-abdominal Aortic Aneurysms in Germany from 2005 to 2014: Secondary Data Analysis of the Nationwide German DRG Microdata. Eur J Vasc Endovasc Surg. 2019 Apr;57(4):488–98.

101. Gershman B, Meier SK, Jeffery MM, Moreira DM, Tollefson MK, Kim SP, et al. Redefining and Contextualizing the Hospital Volume-Outcome Relationship for Robot-Assisted Radical Prostatectomy: Implications for Centralization of Care. Journal of Urology. 2017 Jul;198(1):92–9.

102. Ghaferi AA, Birkmeyer JD, Dimick JB. Hospital volume and failure to rescue with high-risk surgery. Med Care. 2011 Dec;49(12):1076–81.

103. Gietelink L, Henneman D, van Leersum NJ, de Noo M, Manusama E, Tanis PJ, et al. The Influence of Hospital Volume on Circumferential Resection Margin Involvement: Results of the Dutch Surgical Colorectal Audit. Annals of Surgery. 2016 Apr;263(4):745–50.

104. Glassou EN, Hansen TB, Mäkelä K, Havelin LI, Furnes O, Badawy M, et al. Association between hospital procedure volume and risk of revision after total hip arthroplasty: a population-based study within the Nordic Arthroplasty Register Association database. Osteoarthritis and Cartilage. 2016 Mar;24(3):419–26.

105. Gonzalez AA, Dimick JB, Birkmeyer JD, Ghaferi AA. Understanding the Volume-Outcome Effect in Cardiovascular Surgery: The Role of Failure to Rescue. JAMA Surg. 2014 Feb 1;149(2):119.

106. Gonzalez AA, Sutzko DC, Osborne NH. A National Study Evaluating Hospital Volume and Inpatient Mortality after Open Abdominal Aortic Aneurysm Repair in Vulnerable Populations. Annals of Vascular Surgery. 2018 Jul;50:154–9.

107. Gooiker GA, Lemmens VEPP, Besselink MG, Busch OR, Bonsing BA, Molenaar IQ, et al. Impact of centralization of pancreatic cancer surgery on resection rates and survival: Impact of centralization of pancreatic cancer surgery. Br J Surg. 2014 Jul;101(8):1000–5.

108. Goossens-Laan CA, Visser O, Hulshof MCCM, Wouters MW, Bosch JLHR, Coebergh J-WW, et al. Survival after treatment for carcinoma invading bladder muscle: a Dutch population-based study on the impact of hospital volume: IMPACT OF HOSPITAL VOLUME ON SURVIVAL AFTER BLADDER CANCER. BJU International. 2012 Jul;110(2):226–32.

109. Gort M, Otter R, Plukker JThM, Broekhuis M, Klazinga NS. Actionable indicators for short and long term outcomes in rectal cancer. European Journal of Cancer. 2010 Jul 1;46(10):1808–14.

110. Gottlieb-Vedi E, Mattsson F, Lagergren P, Lagergren J. Annual hospital volume of surgery for gastrointestinal cancer in relation to prognosis. Eur J Surg Oncol. 2019 Oct;45(10):1839–46.

111. Gould JC, Kent KC, Wan Y, Rajamanickam V, Leverson G, Campos GM. Perioperative Safety and Volume: Outcomes Relationships in Bariatric Surgery: A Study of 32,000 Patients. Journal of the American College of Surgeons. 2011 Dec;213(6):771–7.

112. Gourin CG, Stewart CM, Frick KD, Fakhry C, Pitman KT, Eisele DW, et al. Association of Hospital Volume With Laryngectomy Outcomes in Patients With Larynx Cancer. JAMA Otolaryngol Head Neck Surg. 2019 01;145(1):62–70.

113. Goyal G, Kommalapati A, Bartley AC, Gunderson TM, Adjei AA, Go RS. Association between hospital volume and mortality of patients with metastatic non-small cell lung cancer. Lung Cancer. 2018;122:214–9.

114. Grushka JR, Laberge J-M, Puligandla P, Skarsgard ED, Canadian Pediatric Surgery Network. Effect of hospital case volume on outcome in congenital diaphragmatic hernia: the experience of the Canadian Pediatric Surgery Network. J Pediatr Surg. 2009 May;44(5):873–6.

115. Güller U, Warschkow R, Ackermann CJ, Schmied BM. Lower hospital volume is associated with higher mortality after oesophageal, gastric, pancreatic and rectal cancer resection. Swiss Med Wkly [Internet]. 2017 Jul 24 [cited 2020 Mar 4];147(2930). Available from: http://doi.emh.ch/smw.2017.14473

116. Gupta PK, Ramanan B, Grossman L, Gupta H, Fang X, MacTaggart JN, et al. Outcomes of Aortic Surgery for Abdominal Aortic Graft Infections. Vasc Endovascular Surg. 2016 May;50(4):256–60.

117. Gutacker N, Bloor K, Cookson R, Gale CP, Maynard A, Pagano D, et al. Hospital Surgical Volumes and Mortality after Coronary Artery Bypass Grafting: Using International Comparisons to Determine a Safe Threshold. Health Serv Res. 2017 Apr;52(2):863–78.

118. Gutierrez JC, Koniaris LG, Cheung MC, Byrne MM, Fischer AC, Sola JE. Cancer care in the pediatric surgical patient: a paradigm to abolish volume-outcome disparities in surgery. Surgery. 2009 Jan;145(1):76–85.

119. Hagemans JAW, Alberda WJ, Verstegen M, de Wilt JHW, Verhoef C, Elferink MA, et al. Hospital volume and outcome in rectal cancer patients; results of a population-based study in the Netherlands. European Journal of Surgical Oncology. 2019 Apr;45(4):613–9.

120. Hanchanale VS, Javlé P. Impact of Hospital Provider Volume on Outcome for Radical Urological Cancer Surgery in England. Urol Int. 2010;85(1):11–5.

121. Harrison EM, O’Neill S, Meurs TS, Wong PL, Duxbury M, Paterson-Brown S, et al. Hospital volume and patient outcomes after cholecystectomy in Scotland: retrospective, national population based study. BMJ. 2012 May 23;344(may23 1):e3330–e3330.

122. Hatch JL, Bauschard MJ, Nguyen SA, Lambert PR, Meyer TA, McRackan TR. Does Hospital Volume Affect Outcomes in Patients Undergoing Vestibular Schwannoma Surgery? Otol Neurotol. 2018;39(4):481–7.

123. Healy MA, Krell RW, Abdelsattar ZM, McCahill LE, Kwon D, Frankel TL, et al. Pancreatic Resection Results in a Statewide Surgical Collaborative. Ann Surg Oncol. 2015 Aug;22(8):2468–74.

124. Henneman D, van Leersum NJ, Ten Berge M, Snijders HS, Fiocco M, Wiggers T, et al. Failure-to-rescue after colorectal cancer surgery and the association with three structural hospital factors. Ann Surg Oncol. 2013 Oct;20(11):3370–6.

125. Hernandez-Meza G, McKee S, Carlton D, Yang A, Govindaraj S, Iloreta A. Association of Surgical and Hospital Volume and Patient Characteristics With 30-Day Readmission Rates. JAMA Otolaryngol Head Neck Surg. 2019 Apr 1;145(4):328–37.

126. Hjelle KM, Johannesen TB, Beisland C. Postoperative 30-day Mortality Rates for Kidney Cancer Are Dependent on Hospital Surgical Volume: Results from a Norwegian Population-based Study. European Urology Focus. 2017 Apr;3(2–3):300–7.

127. Holt PJE, Karthikesalingam A, Hofman D, Poloniecki JD, Hinchliffe RJ, Loftus IM, et al. Provider volume and long-term outcome after elective abdominal aortic aneurysm repair. Br J Surg. 2012 May;99(5):666–72.

128. Holt PJE, Poloniecki JD, Khalid U, Hinchliffe RJ, Loftus IM, Thompson MM. Effect of endovascular aneurysm repair on the volume-outcome relationship in aneurysm repair. Circ Cardiovasc Qual Outcomes. 2009 Nov;2(6):624–32.

129. Hsu P-K, Chen H-S, Wu S-C, Wang B-Y, Liu C-Y, Shih C-H, et al. Impact of hospital volume on long-term survival after resection for oesophageal cancer: a population-based study in Taiwan†. European Journal of Cardio-Thoracic Surgery. 2014 Dec 1;46(6):e127–35.

130. Hsu RCJ, Barclay M, Loughran MA, Lyratzopoulos G, Gnanapragasam VJ, Armitage JN. Impact of hospital nephrectomy volume on intermediate- to long-term survival in renal cell carcinoma: Long-term RCC volume-outcome relationship. BJU Int. 2020 Jan;125(1):56–63.

131. Hughes GC, Zhao Y, Rankin JS, Scarborough JE, O’Brien S, Bavaria JE, et al. Effects of institutional volumes on operative outcomes for aortic root replacement in North America. J Thorac Cardiovasc Surg. 2013 Jan;145(1):166–70.

132. Huguet M, Perrier L, Bally O, Benayoun D, De Saint Hilaire P, Beal Ardisson D, et al. Being treated in higher volume hospitals leads to longer progression-free survival for epithelial ovarian carcinoma patients in the Rhone-Alpes region of France. BMC Health Serv Res. 2018 Dec;18(1):3.

133. Hyder O, Dodson RM, Nathan H, Schneider EB, Weiss MJ, Cameron JL, et al. Influence of Patient, Physician, and Hospital Factors on 30-Day Readmission Following Pancreatoduodenectomy in the United States. JAMA Surg. 2013 Dec 1;148(12):1095.

134. Ichikawa D, Komatsu S, Kubota T, Okamoto K, Deguchi K, Tamai H, et al. Effect of hospital volume on long-term outcomes of laparoscopic gastrectomy for clinical stage I gastric cancer. Anticancer Res. 2013 Nov;33(11):5165–70.

135. Ikoma N, Kim B, Elting LS, Shih Y-CT, Badgwell BD, Mansfield P. Trends in Volume–Outcome Relationship in Gastrectomies in Texas. Ann Surg Oncol. 2019 Sep;26(9):2694–702.

136. Isogai T, Yasunaga H, Matsui H, Ueda T, Tanaka H, Horiguchi H, et al. Hospital Volume and Cardiac Complications of Endomyocardial Biopsy: A Retrospective Cohort Study of 9508 Adult Patients Using a Nationwide Inpatient Database in Japan: Hospital volume and complications of EMB. Clin Cardiol. 2015 Mar;38(3):164–70.

137. Jalbert JJ, Gerhard-Herman MD, Nguyen LL, Jaff MR, Kumamaru H, Williams LA, et al. Relationship Between Physician and Hospital Procedure Volume and Mortality After Carotid Artery Stenting Among Medicare Beneficiaries. Circ Cardiovasc Qual Outcomes. 2015 Oct;8(6 suppl 3):S81–9.

138. James TA, Kasumova G, Alapati A, Mamtani A. Unplanned readmissions following breast cancer surgery. The American Journal of Surgery. 2019 Nov;218(5):988–92.

139. Jean RA, O’Neill KM, Pei KY, Davis KA. Impact of hospital volume on outcomes for laparoscopic adhesiolysis for small bowel obstruction. Journal of Surgical Research. 2017 Jun;214:23–31.

140. Jeschke E, Citak M, Günster C, Matthias Halder A, Heller K-D, Malzahn J, et al. Are TKAs Performed in High-volume Hospitals Less Likely to Undergo Revision Than TKAs Performed in Low-volume Hospitals? Clin Orthop Relat Res. 2017 Nov;475(11):2669–74.

141. Jeschke E, Gehrke T, Günster C, Heller K-D, Leicht H, Malzahn J, et al. Low Hospital Volume Increases Revision Rate and Mortality Following Revision Total Hip Arthroplasty: An Analysis of 17,773 Cases. The Journal of Arthroplasty. 2019 Sep;34(9):2045–50.

142. Jonker FHW, Hagemans JAW, Verhoef C, Burger JWA. The impact of hospital volume on perioperative outcomes of rectal cancer. European Journal of Surgical Oncology (EJSO). 2017 Oct;43(10):1894–900.

143. Jonker FHW, Hagemans JAW, Burger JWA, Verhoef C, Borstlap WAA, Tanis PJ. The influence of hospital volume on long-term oncological outcome after rectal cancer surgery. Int J Colorectal Dis. 2017 Dec;32(12):1741–7.

144. Juillard C, Lashoher A, Sewell CA, Uddin S, Griffith JG, Chang DC. A National Analysis of the Relationship Between Hospital Volume, Academic Center Status, and Surgical Outcomes for Abdominal Hysterectomy Done for Leiomyoma. Journal of the American College of Surgeons. 2009 Apr 1;208(4):599–606.

145. Juo Y-Y, Sanaiha Y, Khrucharoen U, Tillou A, Dutson E, Benharash P. Complete Impact of Care Fragmentation on Readmissions Following Urgent Abdominal Operations. J Gastrointest Surg. 2019 Aug;23(8):1643–51.

146. Kadlec AO, Ellimoottil C, Guo R, Trinh Q-D, Sun M, Turk TM. Contemporary Volume–Outcome Relationships for Percutaneous Nephrolithotomy: Results from the Nationwide Inpatient Sample. Journal of Endourology. 2013 Sep;27(9):1107–13.

147. Kagedan DJ, Goyert N, Li Q, Paszat L, Kiss A, Earle CC, et al. The Impact of Increasing Hospital Volume on 90-Day Postoperative Outcomes Following Pancreaticoduodenectomy. J Gastrointest Surg. 2017 Mar;21(3):506–15.

148. Kalaitzakis E, Toth E. Hospital Volume Status Is Related to Technical Failure and All-Cause Mortality Following ERCP for Benign Disease. Dig Dis Sci. 2015 Jun;60(6):1793–800.

149. Kalakoti P, Missios S, Menger R, Kukreja S, Konar S, Nanda A. Association of risk factors with unfavorable outcomes after resection of adult benign intradural spine tumors and the effect of hospital volume on outcomes: an analysis of 18, 297 patients across 774 US hospitals using the National Inpatient Sample (2002-2011). Neurosurg Focus. 2015 Aug;39(2):E4.

150. Kamei J, Yazawa S, Yamamoto S, Kaburaki N, Takahashi S, Takeyama M, et al. Risk factors for surgical site infection after transvaginal mesh placement in a nationwide Japanese cohort. Neurourology and Urodynamics. 2018 Mar;37(3):1074–81.

151. Kane JM, Harbert J, Hohmann S, Pillai S, Behal R, Selip D, et al. Case Volume and Outcomes of Congenital Diaphragmatic Hernia Surgery in Academic Medical Centers. Am J Perinatol. 2015 Jul;32(9):845–52.

152. Kanhere HA, Trochsler MI, Kanhere MH, Lord AN, Maddern GJ. Pancreaticoduodenectomy: Outcomes in a Low-Volume, Specialised Hepato Pancreato Biliary Unit. World J Surg. 2014 Jun;38(6):1484–90.

153. Katz DF, Turakhia MP, Sauer WH, Tzou WS, Heath RR, Zipse MM, et al. Safety of Ventricular Tachycardia Ablation in Clinical Practice: Findings from 9699 Hospital Discharge Records. Circ Arrhythm Electrophysiol. 2015 Apr;8(2):362–70.

154. Keller DS, Hashemi L, Lu M, Delaney CP. Short-Term Outcomes for Robotic Colorectal Surgery by Provider Volume. Journal of the American College of Surgeons. 2013 Dec;217(6):1063-1069.e1.

155. Keung EZ, Chiang Y, Cormier JN, Torres KE, Hunt KK, Feig BW, et al. Treatment at low‐volume hospitals is associated with reduced short‐term and long‐term outcomes for patients with retroperitoneal sarcoma. Cancer. 2018 Dec;124(23):4495–503.

156. Khatod M, Cafri G, Namba RS, Inacio MCS, Paxton EW. Risk Factors for Total Hip Arthroplasty Aseptic Revision. The Journal of Arthroplasty. 2014 Jul;29(7):1412–7.

157. Khera R, Pandey A, Koshy T, Ayers C, Nallamothu BK, Das SR, et al. Role of Hospital Volumes in Identifying Low-Performing and High-Performing Aortic and Mitral Valve Surgical Centers in the United States. JAMA Cardiol. 2017 Dec 1;2(12):1322.

158. Khera S, Kolte D, Gupta T, Goldsweig A, Velagapudi P, Kalra A, et al. Association Between Hospital Volume and 30-Day Readmissions Following Transcatheter Aortic Valve Replacement. JAMA Cardiol. 2017 Jul 1;2(7):732.

159. Kim C-G, Kwak EK, Lee S. The relationship between hospital volume and outcome of gastrointestinal cancer surgery in Korea. J Surg Oncol. 2011 Aug 1;104(2):116–23.

160. Kim EY, Song KY, Lee J. Does Hospital Volume Really Affect the Surgical and Oncological Outcomes of Gastric Cancer in Korea? J Gastric Cancer. 2017 Sep;17(3):246–54.

161. Kim LK, Looser P, Swaminathan RV, Minutello RM, Wong SC, Girardi L, et al. Outcomes in patients undergoing coronary artery bypass graft surgery in the United States based on hospital volume, 2007 to 2011. The Journal of Thoracic and Cardiovascular Surgery. 2016 Jun;151(6):1686–92.

162. Kim LK, Swaminathan RV, Looser P, Minutello RM, Wong SC, Bergman G, et al. Hospital Volume Outcomes After Septal Myectomy and Alcohol Septal Ablation for Treatment of Obstructive Hypertrophic Cardiomyopathy: US Nationwide Inpatient Database, 2003-2011. JAMA Cardiol. 2016 Jun 1;1(3):324.

163. Kim W, Wolff S, Ho V. Measuring the Volume-Outcome Relation for Complex Hospital Surgery. Appl Health Econ Health Policy. 2016 Aug;14(4):453–64.

164. Kim YH, Her AY. Relationship between hospital volume and risk-adjusted mortality rate following percutaneous coronary intervention in Korea, 2003 to 2004. Anadolu Kardiyol Derg [Internet]. 2013 Feb 6 [cited 2020 May 6]; Available from: https://www.journalagent.com/anatoljcardiol/pdfs/AnatolJCardiol_13_3_237_242.pdf

165. Kim Y, Dhar VK, Wima K, Jung AD, Xia BT, Hoehn RS, et al. The center volume–outcome effect in pancreas transplantation: a national analysis. Journal of Surgical Research. 2017 Jun;213:25–31.

166. Kinoshita Y, Sugihara T, Yasunaga H, Matsui H, Ishikawa A, Fujimura T, et al. Hospital-Volume Effects on Perioperative Outcomes in Peritoneal Dialysis Catheter Implantation: Analysis of 2,505 Cases. Perit Dial Int. 2018 Nov;38(6):419–23.

167. Kohn GP, Galanko JA, Overby DW, Farrell TM. High case volumes and surgical fellowships are associated with improved outcomes for bariatric surgery patients: a justification of current credentialing initiatives for practice and training. J Am Coll Surg. 2010 Jun;210(6):909–18.

168. Kohn GP, Galanko JA, Meyers MO, Feins RH, Farrell TM. National Trends in Esophageal Surgery—Are Outcomes as Good as We Believe? J Gastrointest Surg. 2009 Nov;13(11):1900–12.

169. Kohn GP, Nikfarjam M. The Effect of Surgical Volume and the Provision of Residency and Fellowship Training on Complications of Major Hepatic Resection. J Gastrointest Surg. 2010 Dec;14(12):1981–9.

170. Konety SH, Rosenthal GE, Vaughan-Sarrazin MS. Surgical volume and outcomes of off-pump coronary artery bypass graft surgery: Does it matter? The Journal of Thoracic and Cardiovascular Surgery. 2009 May;137(5):1116-1123.e1.

171. Kontos MC, Wang Y, Chaudhry SI, Vetrovec GW, Curtis J, Messenger J. Lower Hospital Volume Is Associated With Higher In-Hospital Mortality in Patients Undergoing Primary Percutaneous Coronary Intervention for ST-Segment–Elevation Myocardial Infarction: A Report From the NCDR. Circ Cardiovasc Qual Outcomes. 2013 Nov;6(6):659–67.

172. Kopp W, van Meel M, Putter H, Samuel U, Arbogast H, Schareck W, et al. Center Volume Is Associated With Outcome After Pancreas Transplantation Within the Eurotransplant Region. Transplantation. 2017;101(6):1247–53.

173. Kozower BD, Stukenborg GJ. The Relationship Between Hospital Lung Cancer Resection Volume and Patient Mortality Risk: Annals of Surgery. 2011 Dec;254(6):1032–7.

174. Kozower BD, Stukenborg GJ. Hospital esophageal cancer resection volume does not predict patient mortality risk. Ann Thorac Surg. 2012 May;93(5):1690–6; discussion 1696-1698.

175. Krautz C, Nimptsch U, Weber GF, Mansky T, Grützmann R. Effect of Hospital Volume on In-hospital Morbidity and Mortality Following Pancreatic Surgery in Germany: Annals of Surgery. 2018 Mar;267(3):411–7.

176. Kressner M, Bohe M, Cedermark B, Dahlberg M, Damber L, Lindmark G, et al. The Impact of Hospital Volume on Surgical Outcome in Patients with Rectal Cancer: Diseases of the Colon & Rectum. 2009 Sep;52(9):1542–9.

177. Kuehnl A, Tsantilas P, Knappich C, Schmid S, König T, Breitkreuz T, et al. Significant Association of Annual Hospital Volume With the Risk of Inhospital Stroke or Death Following Carotid Endarterectomy but Likely Not After Carotid Stenting: Secondary Data Analysis of the Statutory German Carotid Quality Assurance Database. Circ Cardiovasc Interv [Internet]. 2016 Nov [cited 2020 May 6];9(11). Available from: https://www.ahajournals.org/doi/10.1161/CIRCINTERVENTIONS.116.004171

178. Kulkarni GS, Urbach DR, Austin PC, Fleshner NE, Laupacis A. Impact of provider volume on operative mortality after radical cystectomy in a publicly funded healthcare system. CUAJ. 2013 Dec 5;7(11–12):425.

179. Kumpulainen S, Sankila R, Leminen A, Kuoppala T, Komulainen M, Puistola U, et al. The effect of hospital operative volume, residual tumor and first-line chemotherapy on survival of ovarian cancer — A prospective nation-wide study in Finland. Gynecologic Oncology. 2009 Nov;115(2):199–203.

180. Kutlu OC, Lee JE, Katz MH, Tzeng C-WD, Wolff RA, Varadhachary GR, et al. Open Pancreaticoduodenectomy Case Volume Predicts Outcome of Laparoscopic Approach: A Population-based Analysis. Annals of Surgery. 2018 Mar;267(3):552–60.

181. Kuwabara K. Effect of Hospital Characteristics on the Quality of Laparoscopic Gastrectomy in Japan. Gastroenterol Res [Internet]. 2010 [cited 2020 Mar 24]; Available from: http://www.gastrores.org/index.php/Gastrores/article/view/189

182. Kuwabara K, Matsuda S, Fushimi K, Ishikawa KB, Horiguchi H, Fujimori K. Impact of Hospital Case Volume on the Quality of Laparoscopic Colectomy in Japan. J Gastrointest Surg. 2009 Sep;13(9):1619–26.

183. Kuwabara K, Matsuda S, Fushimi K, Ishikawa KB, Horiguchi H, Fujimori K, et al. Quantitative Assessment of the Advantages of Laparoscopic Gastrectomy and the Impact of Volume-Related Hospital Characteristics on Resource Use and Outcomes of Gastrectomy Patients in Japan: Annals of Surgery. 2011 Jan;253(1):64–70.

184. Langabeer JR, Kim J, Helton J. Exploring the Relationship Between Volume and Outcomes in Hospital Cardiovascular Care: Quality Management in Health Care. 2017;26(3):160–4.

185. Lansdale N, Al-Khafaji N, Green P, Kenny SE. Population-level surgical outcomes for infantile hypertrophic pyloric stenosis. Journal of Pediatric Surgery. 2018 Mar;53(3):540–4.

186. LaPar DJ, Kron IL, Jones DR, Stukenborg GJ, Kozower BD. Hospital Procedure Volume Should Not Be Used as a Measure of Surgical Quality: Annals of Surgery. 2012 Oct;256(4):606–15.

187. LaRiviere CA, McAteer JP, Huaco JA, Garrison MM, Avansino JR, Koepsell TD, et al. Outcomes in pediatric surgery by hospital volume: a population-based comparison. Pediatr Surg Int. 2013 Jun;29(6):561–70.

188. Laucis NC, Chowdhury M, Dasgupta A, Bhattacharyya T. Trend Toward High-Volume Hospitals and the Influence on Complications in Knee and Hip Arthroplasty: The Journal of Bone and Joint Surgery. 2016 May;98(9):707–12.

189. Lee BY, Ha S, Lee YH. Association between volume of surgery for acute hemorrhagic stroke and mortality. Medicine (Baltimore). 2018 Aug;97(35):e12105.

190. Lee JA, Park JH, Lee EJ, Kim SY, Kim Y, Lee SI. High-quality, low-cost gastrectomy care at high-volume hospitals: results from a population-based study in South Korea. Arch Surg. 2011 Aug;146(8):930–6.

191. Lee J-A, Kim S-Y, Park K, Park E-C, Park J-H. Analysis of Hospital Volume and Factors Influencing Economic Outcomes in Cancer Surgery: Results from a Population-based Study in Korea. Osong Public Health Res Perspect. 2017 Feb;8(1):34–46.

192. Leigh Y, Goldacre M, McCulloch P. Surgical specialty, surgical unit volume and mortality after oesophageal cancer surgery. European Journal of Surgical Oncology (EJSO). 2009 Aug;35(8):820–5.

193. Lenzi J, Lombardi R, Gori D, Zanini N, Tedesco D, Masetti M, et al. Impact of Procedure Volumes and Focused Practice on Short-Term Outcomes of Elective and Urgent Colon Cancer Resection in Italy. Moschetta A, editor. PLoS ONE. 2013 May 16;8(5):e64245.

194. Leonard D, Penninckx F, Kartheuser A, Laenen A, Van Eycken E, PROCARE. Effect of hospital volume on quality of care and outcome after rectal cancer surgery. Br J Surg. 2014 Oct;101(11):1475–82.

195. Lieberman-Cribbin W, Galsky M, Casey M, Liu B, Oh W, Flores R, et al. Hospital Centralization Impacts High-Risk Lung and Bladder Cancer Surgical Patients. Cancer Investigation. 2017 Nov 26;35(10):652–61.

196. Liedberg F, Hagberg O, Aljabery F, Gårdmark T, Hosseini A, Jahnson S, et al. Period-specific mean annual hospital volume of radical cystectomy is associated with outcome and perioperative quality of care: a nationwide population-based study. BJU Int. 2019 Sep;124(3):449–56.

197. Lijftogt N, Karthaus EG, Vahl A, van Zwet EW, van der Willik EM, Tollenaar RAEM, et al. Failure to Rescue - a Closer Look at Mortality Rates Has No Added Value for Hospital Comparisons but Is Useful for Team Quality Assessment in Abdominal Aortic Aneurysm Surgery in The Netherlands. Eur J Vasc Endovasc Surg. 2018 Nov;56(5):652–61.

198. Lindekleiv H, Mathiesen EB, Førde OH, Wilsgaard T, Ingebrigtsen T. Hospital volume and 1-year mortality after treatment of intracranial aneurysms: a study based on patient registries in Scandinavia. J Neurosurg. 2015 Sep;123(3):631–7.

199. Lindgren A, Burt S, Bragan Turner E, Meretoja A, Lee J-M, Hemmen TM, et al. Hospital case-volume is associated with case-fatality after aneurysmal subarachnoid hemorrhage. International Journal of Stroke. 2019 Apr;14(3):282–9.

200. Llorente C, Guijarro A, Hernandez V, Fernández-Conejo G, Perez-Fernandez E, Pocock S. Effect of hospital volume on 90-day mortality after radical cystectomy for bladder cancer in Spain. World J Urol [Internet]. 2019 Jul 13 [cited 2020 Mar 24]; Available from: http://link.springer.com/10.1007/s00345-019-02874-9

201. Lu C-C, Chiu C-C, Wang J-J, Chiu Y-H, Shi H-Y. Volume–Outcome Associations after Major Hepatectomy for Hepatocellular Carcinoma: a Nationwide Taiwan Study. J Gastrointest Surg. 2014 Jun;18(6):1138–45.

202. Lu L-C, Shao Y-Y, Kuo RNC, Lin Z-Z, Yeh Y-C, Shau W-Y, et al. Hospital volume of percutaneous radiofrequency ablation is closely associated with treatment outcomes for patients with hepatocellular carcinoma: Hospital Volume of RFA in HCC. Cancer. 2013 Mar 15;119(6):1210–6.

203. Lüchtenborg M, Riaz SP, Coupland VH, Lim E, Jakobsen E, Krasnik M, et al. High Procedure Volume Is Strongly Associated With Improved Survival After Lung Cancer Surgery. JCO. 2013 Jul 29;31(25):3141–6.

204. Maceroli M, Nikkel LE, Mahmood B, Ketz JP, Qiu X, Ciminelli J, et al. Total Hip Arthroplasty for Femoral Neck Fractures: Improved Outcomes With Higher Hospital Volumes. Journal of Orthopaedic Trauma. 2016 Nov;30(11):597–604.

205. Mahmoudi E, Chung KC. Effect of Hospital Volume on Success of Thumb Replantation. J Hand Surg Am. 2017 Feb;42(2):96-103.e5.

206. Mahmoudi E, Lu Y, Chang S-C, Lin C-Y, Wang Y-C, Chang CJ, et al. Association of High-Volume Surgeons Working in High-Volume Hospitals with Cost of Free Flap Surgeries: Plastic and Reconstructive Surgery - Global Open. 2017 Oct;5(10):e1520.

207. Mäkelä KT, Häkkinen U, Peltola M, Linna M, Kröger H, Remes V. The effect of hospital volume on length of stay, re-admissions, and complications of total hip arthroplasty: A population-based register analysis of 72 hospitals and 30,266 replacements. Acta Orthopaedica. 2011 Feb;82(1):20–6.

208. Marx WH, Simon R, OʼNeill P, Shapiro MJ, Cooper AC, Farrell LS, et al. The Relationship Between Annual Hospital Volume of Trauma Patients and In-Hospital Mortality in New York State: The Journal of Trauma: Injury, Infection, and Critical Care. 2011 Aug;71(2):339–46.

209. Matsuo K, Shimada M, Yamaguchi S, Matoda M, Nakanishi T, Kikkawa F, et al. Association of Radical Hysterectomy Surgical Volume and Survival for Early-Stage Cervical Cancer. Obstet Gynecol. 2019 Jun;133(6):1086–98.

210. Matsushima K, Schaefer EW, Won EJ, Armen SB, Indeck MC, Soybel DI. Positive and Negative Volume-Outcome Relationships in the Geriatric Trauma Population. JAMA Surg. 2014 Apr 1;149(4):319.

211. Maurice MJ, Yih JM, Ammori JB, Abouassaly R. Predictors of surgical quality for retroperitoneal sarcoma: Volume matters. J Surg Oncol. 2017 Nov;116(6):766–74.

212. Mayer EK, Bottle A, Darzi AW, Athanasiou T, Vale JA. The volume-mortality relation for radical cystectomy in England: retrospective analysis of hospital episode statistics. BMJ. 2010 Mar 19;340(mar19 2):c1128–c1128.

213. Mayer EK, Bottle A, Aylin P, Darzi AW, Athanasiou T, Vale JA. The volume-outcome relationship for radical cystectomy in England: an analysis of outcomes other than mortality: *VOLUME-OUTCOME RELATIONSHIP FOR RADICAL CYSTECTOMY*. BJU International. 2011 Oct;108(8b):E258–65.

214. McColl RJ, McGahan CE, Cai E, Olson R, Cheung WY, Raval MJ, et al. Impact of hospital volume on quality indicators for rectal cancer surgery in British Columbia, Canada. The American Journal of Surgery. 2017 Feb;213(2):388–94.

215. McIsaac DI, Wijeysundera DN, Huang A, Bryson GL, van Walraven C. Association of the Hospital Volume of Frail Surgical Patients Cared for with Outcomes after Elective, Major Noncardiac Surgery: A Retrospective Population-based Cohort Study. Anesthesiology. 2017;126(4):602–13.

216. McKee SP, Yang A, Gray M, Zeiger J, Bederson JB, Govindaraj S, et al. Intracranial Meningioma Surgery: Value-Based Care Determinants in New York State, 1995–2015. World Neurosurgery. 2018 Oct;118:e731–44.

217. McNeely C, Markwell S, Filson K, Hazelrigg S, Vassileva C. Effect of Hospital Volume on Prosthesis Use and Mortality in Aortic Valve Operations in the Elderly. The Annals of Thoracic Surgery. 2016 Feb;101(2):585–90.

218. Meguid RA, Weiss ES, Chang DC, Brock MV, Yang SC. The effect of volume on esophageal cancer resections: What constitutes acceptable resection volumes for centers of excellence? The Journal of Thoracic and Cardiovascular Surgery. 2009 Jan;137(1):23–9.

219. Mehta A, Varma S, Efron DT, Joseph BA, Lunardi N, Haut ER, et al. Emergency general surgery in geriatric patients: How should we evaluate hospital experience? Journal of Trauma and Acute Care Surgery. 2019 Feb;86(2):189–95.

220. Menendez ME, Ring D. Failure to rescue after proximal femur fracture surgery. J Orthop Trauma. 2015 Mar;29(3):e96-102.

221. Meng R, Bright T, Woodman RJ, Watson DI. Hospital volume versus outcome following oesophagectomy for cancer in Australia and New Zealand. ANZ J Surg. 2019;89(6):683–8.

222. Menger RP, Kalakoti P, Pugely AJ, Nanda A, Sin A. Adolescent idiopathic scoliosis: risk factors for complications and the effect of hospital volume on outcomes. Neurosurg Focus. 2017 Oct;43(4):E3.

223. Merlo AE, Chauhan D, Pettit C, Hong KN, Saunders CR, Chen C, et al. Outcomes following emergent open repair for thoracic aortic dissection are improved at higher volume centers in direct admissions and transfers. J Cardiothorac Surg. 2016 Dec;11(1):118.

224. Mery CM, Moffett BS, Khan MS, Zhang W, Guzmán-Pruneda FA, Fraser CD, et al. Incidence and treatment of chylothorax after cardiac surgery in children: Analysis of a large multi-institution database. The Journal of Thoracic and Cardiovascular Surgery. 2014 Feb;147(2):678-686.e1.

225. Metcalfe D, Salim A, Olufajo O, Gabbe B, Zogg C, Harris MB, et al. Hospital case volume and outcomes for proximal femoral fractures in the USA: an observational study. BMJ Open. 2016 Apr;6(4):e010743.

226. Miyata H, Motomura N, Ueda Y, Tsukihara H, Tabayashi K, Takamoto S. Toward quality improvement of thoracic aortic surgery: estimating volume-outcome effect from nationwide survey☆. European Journal of Cardio-Thoracic Surgery. 2009 Sep;36(3):517–21.

227. Møller H, Riaz SP, Holmberg L, Jakobsen E, Lagergren J, Page R, et al. High lung cancer surgical procedure volume is associated with shorter length of stay and lower risks of re-admission and death: National cohort analysis in England. European Journal of Cancer. 2016 Sep;64:32–43.

228. Moxey PW, Hofman D, Hinchliffe RJ, Poloniecki J, Loftus IM, Thompson MM, et al. Volume–Outcome Relationships in Lower Extremity Arterial Bypass Surgery: Annals of Surgery. 2012 Dec;256(6):1102–7.

229. Mroczkowski P, Kube R, Ptok H, Schmidt U, Hac S, Köckerling F, et al. Low-volume centre vs high-volume: the role of a quality assurance programme in colon cancer surgery: Low-volume centre vs high-volume. Colorectal Disease. 2011 Sep;13(9):e276–83.

230. Mueller MG, Ellimootil C, Abernethy MG, Mueller ER, Hohmann S, Kenton K. Colpocleisis: a safe, minimally invasive option for pelvic organ prolapse. Female Pelvic Med Reconstr Surg. 2015 Feb;21(1):30–3.

231. Mukhtar RA, Kattan OM, Harris HW. Variation in Annual Volume at a University Hospital Does Not Predict Mortality for Pancreatic Resections. HPB Surgery. 2008 Feb 25;2008:1–6.

232. Mulvey CL, Pronovost PJ, Gourin CG. Hospital volume and failure to rescue after head and neck cancer surgery. Otolaryngol Head Neck Surg. 2015 May;152(5):783–9.

233. Munasinghe A, Markar SR, Mamidanna R, Darzi AW, Faiz OD, Hanna GB, et al. Is It Time to Centralize High-risk Cancer Care in the United States? Comparison of Outcomes of Esophagectomy Between England and the United States: Annals of Surgery. 2015 Jul;262(1):79–85.

234. Murata A, Matsuda S, Kuwabara K, Fujino Y, Kubo T, Fujimori K, et al. Impact of hospital volume on clinical outcomes of endoscopic biliary drainage for acute cholangitis based on the Japanese administrative database associated with the diagnosis procedure combination system. J Gastroenterol. 2010 Oct;45(10):1090–6.

235. Murata A, Mayumi T, Muramatsu K, Ohtani M, Matsuda S. Effect of hospital volume on outcomes of laparoscopic appendectomy for acute appendicitis: an observational study. J Gastrointest Surg. 2015 May;19(5):897–904.

236. Murata A, Muramatsu K, Ichimiya Y, Kubo T, Fujino Y, Matsuda S. Influence of hospital volume on outcomes of laparoscopic gastrectomy for gastric cancer in patients with comorbidity in Japan. Asian Journal of Surgery. 2015 Jan;38(1):33–9.

237. Murata A, Ohtani M, Muramatsu K, Matsuda S. Association between Hospital Volume and Outcomes of Elderly Patients with Hemorrhagic Peptic Ulcer in Japan: An Observational Study. International Journal of Gerontology. 2016 Mar 1;10(1):6–10.

238. Murata A, Okamoto K, Muramatsu K, Matsuda S. Endoscopic submucosal dissection for gastric cancer: the influence of hospital volume on complications and length of stay. Surg Endosc. 2014 Apr;28(4):1298–306.

239. Murphy MM, Knaus WJ, Ng SC, Hill JS, McPhee JT, Shah SA, et al. Total pancreatectomy: a national study. HPB. 2009 Sep;11(6):476–82.

240. Narendra A, Baade PD, Aitken JF, Fawcett J, Smithers BM. Assessment of hospital characteristics associated with improved mortality following complex upper gastrointestinal cancer surgery in Queensland. ANZ Journal of Surgery. 2019 Nov;89(11):1404–9.

241. Nathens AB, Jurkovich GJ, Maier RV, Grossman DC, MacKenzie EJ, Moore M, et al. Relationship Between Trauma Center Volume and Outcomes. :8.

242. Navarro SM, Frankel WC, Haeberle HS, Billow DG, Ramkumar PN. Evaluation of the volume-value relationship in hip fracture care using evidence-based thresholds. HIP International. 2019 Mar 26;112070001983713.

243. Navarro SM, Frankel WC, Haeberle HS, Ramkumar PN. Fixed and Variable Relationship Models to Define the Volume-Value Relationship in Spinal Fusion Surgery: A Macroeconomic Analysis Using Evidence-Based Thresholds. Neurospine. 2018 Sep;15(3):249–60.

244. Navarro SM, Ramkumar PN, Egger AC, Goodwin RC. Evidence-Based Thresholds for the Volume-Value Relationship in Adolescent Idiopathic Scoliosis: Outcomes and Economies of Scale. Spine Deform. 2018 Apr;6(2):156–63.

245. Nielsen ME, Mallin K, Weaver MA, Palis B, Stewart A, Winchester DP, et al. Association of hospital volume with conditional 90-day mortality after cystectomy: an analysis of the National Cancer Data Base: Hospital volume and conditional 90-day post-cystectomy mortality. BJU Int. 2014 Jul;114(1):46–55.

246. Nieman CL, Stewart CM, Eisele DW, Pronovost PJ, Gourin CG. Frailty, hospital volume, and failure to rescue after head and neck cancer surgery. Laryngoscope. 2018;128(6):1365–70.

247. Nijboer A, Ulrich F, Bechstein WO, Schnitzbauer AA. Volume and outcome relation in German liver transplant centers: what lessons can be learned? Transplant Res. 2014;3(1):5.

248. Nimptsch U, Haist T, Gockel I, Mansky T, Lorenz D. Complex gastric surgery in Germany-is centralization beneficial? Observational study using national hospital discharge data. Langenbecks Arch Surg. 2019 Feb;404(1):93–101.

249. Nimptsch U, Haist T, Krautz C, Grützmann R, Mansky T, Lorenz D. Hospital volume, in-hospital mortality, and failure to rescue in esophageal surgery. Deutsches Aerzteblatt Online [Internet]. 2018 Nov 23 [cited 2020 May 21]; Available from: https://www.aerzteblatt.de/10.3238/arztebl.2018.0793

250. Nishigori T, Miyata H, Okabe H, Toh Y, Matsubara H, Konno H, et al. Impact of hospital volume on risk-adjusted mortality following oesophagectomy in Japan: Hospital volume and mortality following oesophagectomy. Br J Surg. 2016 Dec;103(13):1880–6.

251. Nocon CC, Ajmani GS, Bhayani MK. Association of Facility Volume With Positive Margin Rate in the Surgical Treatment of Head and Neck Cancer. JAMA Otolaryngol Head Neck Surg. 2018 Dec 1;144(12):1090.

252. Nojiri Y, Okamura K, Tanaka Y, Yanaihara H, Sassa N, Hattori R, et al. Influence of hospital surgical volume of radical prostatectomy on quality of perioperative care. Int J Clin Oncol. 2013 Oct;18(5):898–904.

253. Nordenvall C, Myrelid P, Ekbom A, Bottai M, Smedby KE, Olén O, et al. Probability, rate and timing of reconstructive surgery following colectomy for inflammatory bowel disease in Sweden: a population-based cohort study. Colorectal Dis. 2015 Oct;17(10):882–90.

254. O’Mahoney PRA, Yeo HL, Sedrakyan A, Trencheva K, Mao J, Isaacs AJ, et al. Centralization of pancreatoduodenectomy a decade later: Impact of the volume–outcome relationship. Surgery. 2016 Jun;159(6):1528–38.

255. O’Donnell TFX, Boitano LT, Deery SE, Lancaster RT, Siracuse JJ, Schermerhorn ML, et al. Hospital Volume Matters: The Volume–Outcome Relationship in Open Juxtarenal AAA Repair. Annals of Surgery. 2020 Jan;271(1):184–90.

256. Odagiri H, Yasunaga H, Matsui H, Fushimi K, Iizuka T, Kaise M. Hospital volume and the occurrence of bleeding and perforation after colorectal endoscopic submucosal dissection: analysis of a national administrative database in Japan. Dis Colon Rectum. 2015 Jun;58(6):597–603.

257. Odagiri H, Yasunaga H, Matsui H, Matsui S, Fushimi K, Kaise M. Hospital volume and adverse events following esophageal endoscopic submucosal dissection in Japan. Endoscopy. 2017 Apr;49(4):321–6.

258. Ogola GO, Crandall ML, Richter KM, Shafi S. High-volume hospitals are associated with lower mortality among high-risk emergency general surgery patients: Journal of Trauma and Acute Care Surgery. 2018 Sep;85(3):560–5.

259. Ogura K, Yasunaga H, Horiguchi H, Ohe K, Shinoda Y, Tanaka S, et al. Impact of hospital volume on postoperative complications and in-hospital mortality after musculoskeletal tumor surgery: analysis of a national administrative database. J Bone Joint Surg Am. 2013 Sep 18;95(18):1684–91.

260. Ohmann C, Verde PE, Blum K, Fischer B, de Cruppé W, Geraedts M. Two Short-Term Outcomes After Instituting a National Regulation Regarding Minimum Procedural Volumes for Total Knee Replacement: The Journal of Bone and Joint Surgery-American Volume. 2010 Mar;92(3):629–38.

261. Okinaga H, Yasunaga H, Hasegawa K, Fushimi K, Kokudo N. Short-Term Outcomes following Hepatectomy in Elderly Patients with Hepatocellular Carcinoma: An Analysis of 10,805 Septuagenarians and 2,381 Octo- and Nonagenarians in Japan. Liver Cancer. 2018;7(1):55–64.

262. Ono S, Ishimaru M, Matsui H, Fushimi K, Yasunaga H. Effect of Hospital Volume on Outcomes of Surgery for Cleft Lip and Palate. J Oral Maxillofac Surg. 2015 Nov;73(11):2219–24.

263. Opotowsky AR, Landzberg MJ, Kimmel SE, Webb GD. Percutaneous closure of patent foramen ovale and atrial septal defect in adults: The impact of clinical variables and hospital procedure volume on in-hospital adverse events. American Heart Journal. 2009 May;157(5):867–74.

264. Otake H, Yasunaga H, Horiguchi H, Matsutani N, Matsuda S, Ohe K. Impact of Hospital Volume on Chest Tube Duration, Length of Stay, and Mortality After Lobectomy. The Annals of Thoracic Surgery. 2011 Sep;92(3):1069–74.

265. Ouyang D, El-Sayed IH, Yom SS. National trends in surgery for sinonasal malignancy and the effect of hospital volume on short-term outcomes: Trends in Sinonasal Cancer Surgery. The Laryngoscope. 2014 Jul;124(7):1609–14.

266. Ozhathil DK, Li Y, Smith JK, Tseng JF, Saidi RF, Bozorgzadeh A, et al. Effect of centre volume and high donor risk index on liver allograft survival. HPB. 2011 Jul;13(7):447–53.

267. Pagès P-B, Cottenet J, Mariet A-S, Bernard A, Quantin C. In-hospital mortality following lung cancer resection: nationwide administrative database. Eur Respir J. 2016 Jun;47(6):1809–17.

268. Pal N, Axisa B, Yusof S, Newcombe RG, Wemyss-Holden S, Rhodes M, et al. Volume and Outcome for Major Upper GI Surgery in England. J Gastrointest Surg. 2008 Feb;12(2):353–7.

269. Pamilo KJ, Peltola M, Paloneva J, Mäkelä K, Häkkinen U, Remes V. Hospital volume affects outcome after total knee arthroplasty. Acta Orthop. 2015 Feb;86(1):41–7.

270. Pamilo KJ, Peltola M, Mäkelä K, Häkkinen U, Paloneva J, Remes V. Is hospital volume associated with length of stay, re-admissions and reoperations for total hip replacement? A population-based register analysis of 78 hospitals and 54,505 replacements. Arch Orthop Trauma Surg. 2013 Dec;133(12):1747–55.

271. Panaich SS, Arora S, Badheka A, Kumar V, Maor E, Raphael C, et al. Procedural trends, outcomes, and readmission rates pre-and post-FDA approval for MitraClip from the National Readmission Database (2013-14). Catheter Cardiovasc Interv. 2018 May 1;91(6):1171–81.

272. Pandey AS, Meurer WJ, Chaudhary N, Gemmete JJ, Thompson BG, Morgenstern LB, et al. Intra-arterial Stroke Treatment prior to the Stent-Retriever Era: High Mortality and Lack of Volume–Outcome Association. Journal of Stroke and Cerebrovascular Diseases. 2016 Oct;25(10):2553–8.

273. Paquette IM, Kemp JA, Finlayson SRG. Patient and Hospital Factors Associated With Use of Sphincter-Sparing Surgery for Rectal Cancer: Diseases of the Colon & Rectum. 2010 Feb;53(2):115–20.

274. Parc Y, Reboul-Marty J, Lefevre JH, Shields C, Chafai N, Tiret E. Factors influencing mortality and morbidity following colorectal resection in France. Analysis of a national database (2009-2011). Colorectal Dis. 2016 Feb;18(2):205–13.

275. Park HS, Detterbeck FC, Boffa DJ, Kim AW. Impact of Hospital Volume of Thoracoscopic Lobectomy on Primary Lung Cancer Outcomes. The Annals of Thoracic Surgery. 2012 Feb;93(2):372–9.

276. Pasquali SK, Jacobs JP, He X, Hornik CP, Jaquiss RDB, Jacobs ML, et al. The Complex Relationship Between Center Volume and Outcome in Patients Undergoing the Norwood Operation. The Annals of Thoracic Surgery. 2012 May;93(5):1556–62.

277. Patel HJ, Herbert MA, Drake DH, Hanson EC, Theurer PF, Bell GF, et al. Aortic Valve Replacement: Using a Statewide Cardiac Surgical Database Identifies a Procedural Volume Hinge Point. The Annals of Thoracic Surgery. 2013 Nov;96(5):1560–6.

278. Patel NJ, Badheka AO, Jhamnani S, Panaich SS, Singh V, Patel N, et al. Effect of Hospital Volume on Outcomes of Transcatheter Mitral Valve Repair: An Early US Experience. J Interv Cardiol. 2015 Oct;28(5):464–71.

279. Patel VI, Mukhopadhyay S, Ergul E, Aranson N, Conrad MF, LaMuraglia GM, et al. Impact of hospital volume and type on outcomes of open and endovascular repair of descending thoracic aneurysms in the United States Medicare population. Journal of Vascular Surgery. 2013 Aug;58(2):346–54.

280. Paterson JM, Williams JI, Kreder HJ, Mahomed NN. Provider volumes and early outcomes of primary total joint replacement in Ontario. :9.

281. Paul JC, Lonner BS, Goz V, Weinreb J, Karia R, Toombs CS, et al. Complication rates are reduced for revision adult spine deformity surgery among high-volume hospitals and surgeons. The Spine Journal. 2015 Sep;15(9):1963–72.

282. Peltoniemi P, Peltola M, Hakulinen T, Häkkinen U, Pylkkänen L, Holli K. The Effect of Hospital Volume on the Outcome of Breast Cancer Surgery. Ann Surg Oncol. 2011 Jun;18(6):1684–90.

283. Pezzin LE, Neuner J. Reexamining the Relationship of Breast Cancer Hospital and Surgical Volume to Mortality. Medical Care. 2015;53(12):7.

284. Pohle M, Magheli A, Fischer T, Ralla B, Miller K, Hinz S. Influences of Surgical Volume on Perioperative and Oncological Outcomes Following Radical Prostatectomy. Urol Int. 2018;101(3):256–62.

285. Porter MP, Gore JL, Wright JL. Hospital volume and 90-day mortality risk after radical cystectomy: a population-based cohort study. World J Urol. 2011 Feb;29(1):73–7.

286. Pucciarelli S, Zorzi M, Gennaro N, Marchegiani F, Barina A, Rugge M, et al. Relationship between hospital volume and short-term outcomes: a nationwide population-based study including 75,280 rectal cancer surgical procedures. Oncotarget [Internet]. 2018 Mar 30 [cited 2020 Mar 12];9(24). Available from: http://www.oncotarget.com/fulltext/24699

287. Ramkumar PN, Navarro SM, Haeberle HS, Ricchetti ET, Iannotti JP. Evidence-based thresholds for the volume-value relationship in shoulder arthroplasty: outcomes and economies of scale. J Shoulder Elbow Surg. 2017 Aug;26(8):1399–406.

288. Rana A, Pallister Z, Halazun K, Cotton R, Guiteau J, Nalty CC, et al. Pediatric Liver Transplant Center Volume and the Likelihood of Transplantation. PEDIATRICS. 2015 Jul 1;136(1):e99–107.

289. Reames BN, Ghaferi AA, Birkmeyer JD, Dimick JB. Hospital volume and operative mortality in the modern era. Ann Surg. 2014 Aug;260(2):244–51.

290. Regenbogen SE, Gust C, Birkmeyer JD. Hospital Surgical Volume and Cost of Inpatient Surgery in the Elderly. Journal of the American College of Surgeons. 2012 Dec;215(6):758–65.

291. Ricciardi BF, Liu AY, Qiu B, Myers TG, Thirukumaran CP. What Is the Association Between Hospital Volume and Complications After Revision Total Joint Arthroplasty: A Large-database Study. Clinical Orthopaedics and Related Research. 2019 May;477(5):1221–31.

292. Rolston JD, Englot DJ, Knowlton RC, Chang EF. Rate and complications of adult epilepsy surgery in North America: Analysis of multiple databases. Epilepsy Research. 2016 Aug;124:55–62.

293. Rosales-Velderrain A. National trends in resection of the distal pancreas. WJG. 2012;18(32):4342.

294. Rosero EB, Joshi GP, Minhajuddin A, Timaran CH, Modrall JG. Effects of hospital safety-net burden and hospital volume on failure to rescue after open abdominal aortic surgery. Journal of Vascular Surgery. 2017 Aug;66(2):404–12.

295. Sabir EF, Holmäng S, Liedberg F, Ljungberg B, Malmström P-U, Månsson W, et al. Impact of hospital volume on local recurrence and distant metastasis in bladder cancer patients treated with radical cystectomy in Sweden. Scandinavian Journal of Urology. 2013 Dec;47(6):483–90.

296. Sacks GD, Ulloa JG, Shew SB. Is there a relationship between hospital volume and patient outcomes in gastroschisis repair? Journal of Pediatric Surgery. 2016 Oct;51(10):1650–4.

297. Sakata R, Kuwano H, Yokomise H. Hospital volume and outcomes of cardiothoracic surgery in Japan: 2005–2009 national survey. Gen Thorac Cardiovasc Surg. 2012 Oct;60(10):625–38.

298. Sakhuja A, Kashani K, Schold J, Cheungpasitporn W, Soltesz E, Demirjian S. Hospital procedure volume does not predict acute kidney injury after coronary artery bypass grafting—a nationwide study. Clinical Kidney Journal. 2017 Dec 1;10(6):769–75.

299. Salfity H, Timsina L, Su K, Ceppa D, Birdas T. Case Volume-to-Outcome Relationship in Minimally Invasive Esophagogastrectomy. The Annals of Thoracic Surgery. 2019 Nov;108(5):1491–7.

300. Sammon JD, Karakiewicz PI, Sun M, Sukumar S, Ravi P, Ghani KR, et al. Robot-Assisted Versus Open Radical Prostatectomy: The Differential Effect of Regionalization, Procedure Volume and Operative Approach. Journal of Urology. 2013 Apr;189(4):1289–94.

301. Sammon JD, Klett DE, Sood A, Olugbade K, Schmid M, Kim SP, et al. Sepsis after major cancer surgery. Journal of Surgical Research. 2015 Feb;193(2):788–94.

302. Sanaiha Y, Juo Y-Y, Aguayo E, Seo Y-J, Dobaria V, Ziaeian B, et al. Incidence and trends of cardiac complications in major abdominal surgery. Surgery. 2018 Sep;164(3):539–45.

303. Sanaiha Y, Khoury H, Kavianpour B, Yazdani S, Gowland L, Iyengar A, et al. Impact of Approach and Hospital Volume on Cardiovascular Complications After Pulmonary Lobectomy. Journal of Surgical Research. 2019 Mar;235:202–9.

304. Sarwar A, Zhou L, Novack V, Tapper EB, Curry M, Malik R, et al. Hospital volume and mortality after transjugular intrahepatic portosystemic shunt creation in the United States. Hepatology. 2018 Feb;67(2):690–9.

305. Sato M, Tateishi R, Yasunaga H, Matsui H, Fushimi K, Ikeda H, et al. Association between hospital volume and in-hospital mortality following radiofrequency ablation for hepatocellular carcinoma: Hospital volume and mortality in radiofrequency ablation for hepatocellular carcinoma. BJS Open. 2017 Apr;1(2):50–4.

306. Sawang M, Paravastu SCV, Liu Z, Thomas SD, Beiles CB, Mwipatayi BP, et al. The Relationship Between Aortic Aneurysm Surgery Volume and Peri-Operative Mortality in Australia. European Journal of Vascular and Endovascular Surgery. 2019 Apr;57(4):510–9.

307. Scali ST, Giles KA, Kubilis P, Beck AW, Crippen CJ, Hughes SJ, et al. Impact of hospital volume on patient safety indicators and failure to rescue following open aortic aneurysm repair. Journal of Vascular Surgery. 2020 Apr;71(4):1135-1146.e4.

308. Schneider EB, Ejaz A, Spolverato G, Hirose K, Makary MA, Wolfgang CL, et al. Hospital volume and patient outcomes in hepato-pancreatico-biliary surgery: is assessing differences in mortality enough? J Gastrointest Surg. 2014 Dec;18(12):2105–15.

309. Schneider EB, Hyder O, Wolfgang CL, Dodson RM, Haider AH, Herman JM, et al. Provider versus patient factors impacting hospital length of stay after pancreaticoduodenectomy. Surgery. 2013 Aug;154(2):152–61.

310. Schoenfeld AJ, Ferrone ML, Sturgeon DJ, Harris MB. Volume-Outcome Relationship in Surgical Interventions for Spinal Metastases: The Journal of Bone and Joint Surgery. 2017 Oct;99(20):1753–9.

311. Shahian DM, O’Brien SM, Normand S-LT, Peterson ED, Edwards FH. Association of hospital coronary artery bypass volume with processes of care, mortality, morbidity, and the Society of Thoracic Surgeons composite quality score. The Journal of Thoracic and Cardiovascular Surgery. 2010 Feb;139(2):273–82.

312. Shi H-Y, Chang H-T, Culbertson R, Chen Y-J, Liao Y-C, Hou M-F. Breast cancer surgery volume-cost associations: Hierarchical linear regression and propensity score matching analysis in a nationwide Taiwan population. Surgical Oncology. 2013 Sep;22(3):178–83.

313. Shi H-Y, Hwang S-L, Lee K-T, Lin C-L. Temporal trends and volume-outcome associations after traumatic brain injury: a 12-year study in Taiwan. JNS. 2013 Apr;118(4):732–8.

314. Shi H-Y, Lee K-T, Chiu C-C, Lee H-H. The volume–outcome relationship in laparoscopic cholecystectomy: a population-based study using propensity score matching. Surg Endosc. 2013 Sep;27(9):3139–45.

315. Shi H-Y, Wang S-N, Lee K-T. Temporal trends and volume-outcome associations in periampullary cancer patients: a propensity score–adjusted nationwide population-based study. The American Journal of Surgery. 2014 Apr;207(4):512–9.

316. Shi H-Y, Wang S-N, Wang SC, Chuang S-C, Chen C-M, Lee K-T. Preoperative transarterial chemoembolization and resection for hepatocellular carcinoma: A nationwide Taiwan database analysis of long-term outcome predictors: Outcomes of TACE for Resectable HCC. J Surg Oncol. 2014 Apr;109(5):487–93.

317. Shin KW, Lee HJ, Nam CM, Moon KT, Park E-C. Hospital characteristics related to the hospital length of stay among inpatients receiving invasive cervical discectomy due to road traffic accidents under automobile insurance in South Korea. BMC Health Serv Res. 2017 Dec;17(1):567.

318. Shinjo D, Matsumoto K, Terashima K, Takimoto T, Ohnuma T, Noguchi T, et al. Volume effect in paediatric brain tumour resection surgery: analysis of data from the Japanese national inpatient database. European Journal of Cancer. 2019 Mar;109:111–9.

319. Shuhaiber J, Isaacs AJ, Sedrakyan A. The Effect of Center Volume on In-Hospital Mortality After Aortic and Mitral Valve Surgical Procedures: A Population-Based Study. The Annals of Thoracic Surgery. 2015 Oct;100(4):1340–6.

320. Siemens DR, Mackillop WJ, Peng Y, Berman D, Elharram A, Rhee J, et al. Processes of Care and the Impact of Surgical Volumes on Cancer-specific Survival: A Population-based Study in Bladder Cancer. Urology. 2014 Nov;84(5):1049–57.

321. Siesling S, Tjan-Heijnen VCG, de Roos M, Snel Y, van Dalen T, Wouters MW, et al. Impact of hospital volume on breast cancer outcome: a population-based study in the Netherlands. Breast Cancer Res Treat. 2014 Aug;147(1):177–84.

322. Simunovic M, Urbach D, Major D, Sutradhar R, Baxter N, To T, et al. Assessing the Volume-Outcome Hypothesis and Region-Level Quality Improvement Interventions: Pancreas Cancer Surgery in Two Canadian Provinces. Ann Surg Oncol. 2010 Oct;17(10):2537–44.

323. Singh JA, Kwoh CK, Boudreau RM, Lee G-C, Ibrahim SA. Hospital volume and surgical outcomes after elective hip/knee arthroplasty: a risk-adjusted analysis of a large regional database. Arthritis Rheum. 2011 Aug;63(8):2531–9.

324. Singh JA, Ramachandran R. Does Hospital Volume Predict Outcomes and Complications After Total Shoulder Arthroplasty in the US?: Shoulder Arthroplasty Outcomes Predicted by Hospital Volume. Arthritis Care & Research. 2015 May;67(6):885–90.

325. Skipworth RJE, Parks RW, Stephens NA, Graham C, Brewster DH, Garden OJ, et al. The relationship between hospital volume and post-operative mortality rates for upper gastrointestinal cancer resections: Scotland 1982–2003. European Journal of Surgical Oncology (EJSO). 2010 Feb;36(2):141–7.

326. Smith ME, Sutzko DC, Davis FM, Eliason JL, Henke PK, Osborne NH. Volume Standards for Open Abdominal Aortic Aneurysm Repair Are Not Associated With Improved Clinical Outcomes. Annals of Vascular Surgery. 2020 Jan;62:1–7.

327. Smith RC, Creighton N, Lord RV, Merrett ND, Keogh GW, Liauw WS, et al. Survival, mortality and morbidity outcomes after oesophagogastric cancer surgery in New South Wales, 2001–2008. Medical Journal of Australia. 2014 Apr;200(7):408–13.

328. Sømme S, Shahi N, McLeod L, Torok M, McManus B, Ziegler MM. Neonatal surgery in low- vs. high-volume institutions: a KID inpatient database outcomes and cost study after repair of congenital diaphragmatic hernia, esophageal atresia, and gastroschisis. Pediatr Surg Int. 2019 Nov;35(11):1293–300.

329. Spolverato G, Ejaz A, Hyder O, Kim Y, Pawlik TM. Failure to rescue as a source of variation in hospital mortality after hepatic surgery. Br J Surg. 2014 Jun;101(7):836–46.

330. Spolverato G, Gennaro N, Zorzi M, Rugge M, Mescoli C, Saugo M, et al. Failure to rescue as a source of variation in hospital mortality after rectal surgery: The Italian experience. Eur J Surg Oncol. 2019 Jul;45(7):1219–24.

331. Stavrou E, S. Smith G, Baker DF. Surgical Outcomes Associated with Oesophagectomy in New South Wales: An Investigation of Hospital Volume. J Gastrointest Surg. 2010 Jun;14(6):951–7.

332. Stenberg E, Szabo E, Ågren G, Näslund E, Boman L, Bylund A, et al. Early Complications After Laparoscopic Gastric Bypass Surgery: Results From the Scandinavian Obesity Surgery Registry. Annals of Surgery. 2014 Dec;260(6):1040–7.

333. Stern JR, Sun T, Mao J, Sedrakyan A, Meltzer AJ. A Decade of Thoracic Endovascular Aortic Aneurysm Repair in New York State: Volumes, Outcomes, and Implications for the Dissemination of Endovascular Technology. Annals of Vascular Surgery. 2019 Jan 1;54:123–33.

334. Stevens CL, Watters DAK. Short‐term outcomes of pancreaticoduodenectomy in the state of Victoria: hospital resources are more important than volume. ANZ Journal of Surgery. 2019 Dec;89(12):1577–81.

335. Sugihara T, Yasunaga H, Horiguchi H, Nishimatsu H, Kume H, Matsuda S, et al. Impact of Hospital Volume and Laser Use on Postoperative Complications and In-Hospital Mortality in Cases of Benign Prostate Hyperplasia. Journal of Urology. 2011 Jun;185(6):2248–53.

336. Sukumar S, Djahangirian O, Sood A, Sammon JD, Varda B, Janosek-Albright K, et al. Minimally Invasive vs Open Pyeloplasty in Children: The Differential Effect of Procedure Volume on Operative Outcomes. Urology. 2014 Jul;84(1):180–4.

337. Sun M, Bianchi M, Trinh Q-D, Abdollah F, Schmitges J, Jeldres C, et al. Hospital Volume is a Determinant of Postoperative Complications, Blood Transfusion and Length of Stay After Radical or Partial Nephrectomy. Journal of Urology. 2012 Feb;187(2):405–10.

338. Sun M, Ravi P, Karakiewicz PI, Sukumar S, Sammon J, Bianchi M, et al. Is there a relationship between leapfrog volume thresholds and perioperative outcomes after radical cystectomy? Urologic Oncology: Seminars and Original Investigations. 2014 Jan;32(1):27.e7-27.e13.

339. Sund R. Modeling the volume-effectiveness relationship in the case of hip fracture treatment in Finland. BMC Health Serv Res. 2010 Dec;10(1):238.

340. Sutton JM, Hoehn RS, Ertel AE, Wilson GC, Hanseman DJ, Wima K, et al. Cost-Effectiveness in Hepatic Lobectomy: the Effect of Case Volume on Mortality, Readmission, and Cost of Care. J Gastrointest Surg. 2016 Feb;20(2):253–61.

341. Sutton JM, Wilson GC, Wima K, Hoehn RS, Cutler Quillin R, Hanseman DJ, et al. Readmission After Pancreaticoduodenectomy: The Influence of the Volume Effect Beyond Mortality. Ann Surg Oncol. 2015 Nov;22(12):3785–92.

342. Suzuki H, Gotoh M, Sugihara K, Kitagawa Y, Kimura W, Kondo S, et al. Nationwide survey and establishment of a clinical database for gastrointestinal surgery in Japan: Targeting integration of a cancer registration system and improving the outcome of cancer treatment. Cancer Science. 2011 Jan;102(1):226–30.

343. Tabbutt S, Ghanayem N, Ravishankar C, Sleeper LA, Cooper DS, Frank DU, et al. Risk factors for hospital morbidity and mortality after the Norwood procedure: A report from the Pediatric Heart Network Single Ventricle Reconstruction trial. The Journal of Thoracic and Cardiovascular Surgery. 2012 Oct;144(4):882–95.

344. Tanaka M, Kanemitsu Y, Ueno H, Kobayashi H, Konishi T, Ishida F, et al. Prognostic impact of hospital volume on familial adenomatous polyposis: a nationwide multicenter study. Int J Colorectal Dis. 2017 Oct;32(10):1489–98.

345. Tanna N, Clayton JL, Roostaeian J, Perry AD, Crisera CA. The Volume-Outcome Relationship for Immediate Breast Reconstruction: Plastic and Reconstructive Surgery. 2012 Jan;129(1):19–24.

346. Tchouta LN, Park HS, Boffa DJ, Blasberg JD, Detterbeck FC, Kim AW. Hospital Volume and Outcomes of Robot-Assisted Lobectomies. Chest. 2017 Feb;151(2):329–39.

347. Teh SH. Patient and Hospital Characteristics on the Variance of Perioperative Outcomes for Pancreatic Resection in the United States: A Plea for Outcome-Based and Not Volume-Based Referral Guidelines. Arch Surg. 2009 Aug 14;144(8):713.

348. Thai AA, Stuart E, Te Marvelde L, Milne RL, Knight S, Whitfield K, et al. Hospital lung surgery volume and patient outcomes. Lung Cancer. 2019;129:22–7.

349. Titsworth WL, Scott RM, Smith ER. National Analysis of 2454 Pediatric Moyamoya Admissions and the Effect of Hospital Volume on Outcomes. Stroke. 2016 May;47(5):1303–11.

350. Tom CM, Niino C, Lee AD, Friedlander S, Sakai-Bizmark R, Lee SL. Effects of Hospital Volume on Patient Outcomes and Costs in Infants With Pyloric Stenosis. J Surg Res. 2019;233:65–73.

351. Toren P, Abouassaly R, Timilshina N, Kulkarni G, Alibhai S, Finelli A. Results of a National Population-based Study of Outcomes of Surgery for Renal Tumors Associated With Inferior Vena Cava Thrombus. Urology. 2013 Sep;82(3):572–8.

352. Tracy ET, Bennett KM, Aviki EM, Pappas TN, Collins BH, Tuttle-Newhall JE, et al. Temporal trends in liver transplant centre volume in the USA. HPB. 2009 Aug;11(5):414–21.

353. Tracy ET, Bennett KM, Danko ME, Diesen DL, Westmoreland TJ, Kuo PC, et al. Low volume is associated with worse patient outcomes for pediatric liver transplant centers. Journal of Pediatric Surgery. 2010 Jan;45(1):108–13.

354. Trenner M, Haller B, Söllner H, Storck M, Umscheid T, Niedermeier H, et al. Twelve years of the quality assurance registry on ruptured and non-ruptured abdominal aortic aneurysms of the German Vascular Society (DGG): Part 3: Predictors of perioperative outcome with a focus on annual caseload. English version. Gefässchirurgie. 2015 Jan;20(S1):32–44.

355. Trenner M, Kuehnl A, Salvermoser M, Reutersberg B, Geisbuesch S, Schmid V, et al. Editor’s Choice - High Annual Hospital Volume is Associated with Decreased in Hospital Mortality and Complication Rates Following Treatment of Abdominal Aortic Aneurysms: Secondary Data Analysis of the Nationwide German DRG Statistics from 2005 to 2013. Eur J Vasc Endovasc Surg. 2018;55(2):185–94.

356. Trinh Q-D, Bianchi M, Hansen J, Tian Z, Abdollah F, Shariat SF, et al. In-hospital Mortality and Failure to Rescue After Cytoreductive Nephrectomy. European Urology. 2013 Jun;63(6):1107–14.

357. Trinh Q-D, Sun M, Sammon J, Tian Z, Bianchi M, Shariat SF, et al. Leapfrog volume thresholds and perioperative complications after radical prostatectomy: Leapfrog Thresholds and Prostatectomy. Cancer. 2012 Oct 15;118(20):4991–8.

358. Trinh VQ, Ravi P, Abd-El-Barr A-E-RM, Jhaveri JK, Gervais M-K, Meyer CP, et al. Pneumonia after Major Cancer Surgery: Temporal Trends and Patterns of Care. Canadian Respiratory Journal. 2016;2016:1–7.

359. Tuggle CT, Patel A, Broer N, Persing JA, Sosa JA, Au AF. Increased hospital volume is associated with improved outcomes following abdominal-based breast reconstruction. Journal of Plastic Surgery and Hand Surgery. 2014 Dec;48(6):382–8.

360. Udovicich C, Perera M, Huq M, Wong L-M, Lenaghan D. Hospital volume and perioperative outcomes for radical cystectomy: a population study. BJU Int. 2017 May;119:26–32.

361. Uppal S, Spencer RJ, Rice LW, Del Carmen MG, Reynolds RK, Griggs JJ. Hospital Readmission as a Poor Measure of Quality in Ovarian Cancer Surgery. Obstet Gynecol. 2018;132(1):126–36.

362. Vakili BA, Kaplan R, Brown DL. Volume-Outcome Relation for Physicians and Hospitals Performing Angioplasty for Acute Myocardial Infarction in New York State. :7.

363. van der Geest LGM, van Rijssen LB, Molenaar IQ, de Hingh IH, Groot Koerkamp B, Busch ORC, et al. Volume–outcome relationships in pancreatoduodenectomy for cancer. HPB. 2016 Apr;18(4):317–24.

364. van der Werf LR, Cords C, Arntz I, Belt EJT, Cherepanin IM, Coene P-PLO, et al. Population-Based Study on Risk Factors for Tumor-Positive Resection Margins in Patients with Gastric Cancer. Ann Surg Oncol. 2019 Jul;26(7):2222–33.

365. van Erning FN, van Steenbergen LN, van den Broek WT, Rutten HJT, Lemmens VEPP. No difference between lowest and highest volume hospitals in outcome after colorectal cancer surgery in the southern Netherlands. European Journal of Surgical Oncology (EJSO). 2013 Nov;39(11):1199–206.

366. van Rijssen LB, Zwart MJ, van Dieren S, de Rooij T, Bonsing BA, Bosscha K, et al. Variation in hospital mortality after pancreatoduodenectomy is related to failure to rescue rather than major complications: a nationwide audit. HPB. 2018 Aug;20(8):759–67.

367. Varagunam M, Hutchings A, Black N. Relationship Between Patient-reported Outcomes of Elective Surgery and Hospital and Consultant Volume: Medical Care. 2015 Feb;1.

368. Varban OA, Reames BN, Finks JF, Thumma JR, Dimick JB. Hospital volume and outcomes for laparoscopic gastric bypass and adjustable gastric banding in the modern era. Surg Obes Relat Dis. 2015 Apr;11(2):343–9.

369. Varghese TK, Wood DE, Farjah F, Oelschlager BK, Symons RG, MacLeod KE, et al. Variation in Esophagectomy Outcomes in Hospitals Meeting Leapfrog Volume Outcome Standards. The Annals of Thoracic Surgery. 2011 Apr;91(4):1003–10.

370. Vassileva CM, McNeely C, Spertus J, Markwell S, Hazelrigg S. Hospital volume, mitral repair rates, and mortality in mitral valve surgery in the elderly: an analysis of US hospitals treating Medicare fee-for-service patients. J Thorac Cardiovasc Surg. 2015 Mar;149(3):762-768.e1.

371. Vemulapalli S, Carroll JD, Mack MJ, Li Z, Dai D, Kosinski AS, et al. Procedural Volume and Outcomes for Transcatheter Aortic-Valve Replacement. N Engl J Med. 2019 Jun 27;380(26):2541–50.

372. Villar JM, Moreno P, Ortega J, Bollo E, Ramírez CP, Muñoz N, et al. Results of adrenal surgery. Data of a Spanish National Survey. Langenbecks Arch Surg. 2010 Sep;395(7):837–43.

373. Vogel TR, Dombrovskiy VY, Graham AM. Carotid Artery Stenting in the Nation: The Influence of Hospital and Physician Volume on Outcomes. Vasc Endovascular Surg. 2010 Feb;44(2):89–94.

374. Vogel TR, Dombrovskiy VY, Graham AM, Lowry SF. The Impact of Hospital Volume on the Development of Infectious Complications After Elective Abdominal Aortic Surgery in the Medicare Population. Vasc Endovascular Surg. 2011 May;45(4):317–24.

375. Wada T, Yasunaga H, Doi K, Matsui H, Fushimi K, Kitsuta Y, et al. Impact of hospital volume on mortality in patients with severe torso injury. Journal of Surgical Research. 2018 Feb;222:1–9.

376. Wakeam E, Hyder JA, Lipsitz SR, Darling GE, Finlayson SRG. Outcomes and Costs for Major Lung Resection in the United States: Which Patients Benefit Most From High-Volume Referral? The Annals of Thoracic Surgery. 2015 Sep;100(3):939–46.

377. Wallenstein MR, Ananth CV, Kim JH, Burke WM, Hershman DL, Lewin SN, et al. Effect of surgical volume on outcomes for laparoscopic hysterectomy for benign indications. Obstet Gynecol. 2012 Apr;119(4):709–16.

378. Ward BK, Gourin CG, Francis HW. Vestibular Schwannoma Surgical Volume and Short-term Outcomes in Maryland. Arch Otolaryngol Head Neck Surg. 2012 Jun 1;138(6):577.

379. Wasif N, Etzioni D, Habermann EB, Mathur A, Chang Y-H. Contemporary Improvements in Postoperative Mortality After Major Cancer Surgery are Associated with Weakening of the Volume-Outcome Association. Ann Surg Oncol. 2019 Aug;26(8):2348–56.

380. Wassef AWA, Rodes-Cabau J, Liu Y, Webb JG, Barbanti M, Muñoz-García AJ, et al. The Learning Curve and Annual Procedure Volume Standards for Optimum Outcomes of Transcatheter Aortic Valve Replacement. JACC: Cardiovascular Interventions. 2018 Sep;11(17):1669–79.

381. Weiss A, Anderson JA, Green A, Chang DC, Kansal N. Hospital volume of thoracoabdominal aneurysm repair does not affect mortality in California. Vasc Endovascular Surg. 2014 Aug;48(5–6):378–82.

382. Wes AM, Mazzaferro D, Naran S, Hopkins E, Bartlett SP, Taylor JA. Craniosynostosis Surgery: Does Hospital Case Volume Impact Outcomes or Cost? Plastic and Reconstructive Surgery. 2017 Nov;140(5):711e–8e.

383. Whealon MD, Blondet JJ, Gahagan JV, Phelan MJ, Nguyen NT. Volume and outcomes relationship in laparoscopic diaphragmatic hernia repair. Surg Endosc. 2017 Oct;31(10):4224–30.

384. Wright JD, Chen L, Buskwofie A, Tergas AI, St. Clair CM, Hou JY, et al. Regionalization of care for women with ovarian cancer. Gynecologic Oncology. 2019 Aug;154(2):394–400.

385. Wright JD, Hershman DL, Burke WM, Lu Y-S, Neugut AI, Lewin SN, et al. Influence of Surgical Volume on Outcome for Laparoscopic Hysterectomy for Endometrial Cancer. Ann Surg Oncol. 2012 Mar;19(3):948–58.

386. Wright JD, Herzog TJ, Siddiq Z, Arend R, Neugut AI, Burke WM, et al. Failure to rescue as a source of variation in hospital mortality for ovarian cancer. J Clin Oncol. 2012 Nov 10;30(32):3976–82.

387. Wright JD, Lewin SN, Deutsch I, Burke WM, Sun X, Herzog TJ. Effect of Surgical Volume on Morbidity and Mortality of Abdominal Hysterectomy for Endometrial Cancer: Obstetrics & Gynecology. 2011 May;117(5):1051–9.

388. Wright JD, Lewin SN, Deutsch I, Burke WM, Sun X, Herzog TJ. The influence of surgical volume on morbidity and mortality of radical hysterectomy for cervical cancer. American Journal of Obstetrics and Gynecology. 2011 Sep;205(3):225.e1-225.e7.

389. Wright JD, Ruiz MP, Chen L, Gabor LR, Tergas AI, St. Clair CM, et al. Changes in Surgical Volume and Outcomes Over Time for Women Undergoing Hysterectomy for Endometrial Cancer: Obstetrics & Gynecology. 2018 Jul;132(1):59–69.

390. Wu J-M, Ho T-W, Tien Y-W. Correlation Between the Increased Hospital Volume and Decreased Overall Perioperative Mortality in One Universal Health Care System. World J Surg. 2019;43(9):2194–202.

391. Wu RT, Shultz BN, Peck CJ, Smetona JT, Steinbacher DM. Hospital Volume Improves Primary, Revision, and Delayed Cleft Palate Repair. J Craniofac Surg. 2019 Jun;30(4):1201–5.

392. Xia L, Pulido JE, Chelluri RR, Strother MC, Taylor BL, Raman JD, et al. Hospital volume and outcomes of robot-assisted partial nephrectomy. BJU Int. 2018;121(6):900–7.

393. Xia L, Strother MC, Taylor BL, Chelluri RR, Pulido JE, Guzzo TJ. Hospital volume and short-term outcomes after cytoreductive nephrectomy. J Surg Oncol. 2018 Jun;117(7):1589–96.

394. Xia L, Taylor BL, Mamtani R, Christodouleas JP, Guzzo TJ. Associations Between Travel Distance, Hospital Volume, and Outcomes Following Radical Cystectomy in Patients With Muscle-invasive Bladder Cancer. Urology. 2018 Apr;114:87–94.

395. Yamaguchi T, Nakai M, Sumita Y, Nishimura K, Miyamoto T, Sakata Y, et al. The impact of institutional case volume on the prognosis of ruptured aortic aneurysms: a Japanese nationwide study. Interactive CardioVascular and Thoracic Surgery. 2019 Jul 1;29(1):109–16.

396. Yasunaga H, Horiguchi H, Matsuda S, Fushimi K, Hashimoto H, Ohe K, et al. Relationship between hospital volume and operative mortality for liver resection: Data from the Japanese Diagnosis Procedure Combination database: Volumes and outcomes in liver resection. Hepatology Research. 2012 Nov;42(11):1073–80.

397. Yasunaga H, Matsuyama Y, Ohe K. Volume-outcome relationship in rectal cancer surgery: A new perspective. Surg Today. 2009 Aug;39(8):663–8.

398. Yasunaga H, Tsuchiya K, Matsuyama Y, Ohe K. Analysis of factors affecting operating time, postoperative complications, and length of stay for total knee arthroplasty: nationwide web-based survey. J Orthop Sci. 2009 Jan;14(1):10–6.

399. Yasunaga H, Yanaihara H, Fuji K, Horiguchi H, Hashimoto H, Matsuda S. Impact of Hospital Volume on Postoperative Complications and In-hospital Mortality After Renal Surgery: Data From the Japanese Diagnosis Procedure Combination Database. Urology. 2010 Sep;76(3):548–52.

400. Yeo I, Kim LK, Wong SC, Cheung JW, Itagaki S, Chikwe J, et al. Relation of Hospital Volume With In-Hospital and 90-Day Outcomes After Transcatheter Mitral Valve Repair Using MitraClip. The American Journal of Cardiology. 2019 Jul;124(1):63–9.

401. Yoshioka R, Yasunaga H, Hasegawa K, Horiguchi H, Fushimi K, Aoki T, et al. Impact of hospital volume on hospital mortality, length of stay and total costs after pancreaticoduodenectomy: Outcome-volume relationship for pancreaticoduodenectomy. Br J Surg. 2014 Apr;101(5):523–9.

402. Yu H-Y, Hevelone ND, Lipsitz SR, Kowalczyk KJ, Nguyen PL, Hu JC. Hospital volume, utilization, costs and outcomes of robot-assisted laparoscopic radical prostatectomy. J Urol. 2012 May;187(5):1632–7.

403. Yu T-H, Tung Y-C, Chung K-P. Does Categorization Method Matter in Exploring Volume-Outcome Relation? A Multiple Categorization Methods Comparison in Coronary Artery Bypass Graft Surgery Surgical Site Infection. Surg Infect (Larchmt). 2015 Aug;16(4):466–72.
